# HsTRPA of the Red Imported Fire Ant, *Solenopsis invicta*, Functions as a Nocisensor and Uncovers the Evolutionary Plasticity of HsTRPA Channels

**DOI:** 10.1523/ENEURO.0327-17.2018

**Published:** 2018-02-06

**Authors:** Xinyue Wang, Tianbang Li, Makiko Kashio, Yijuan Xu, Makoto Tominaga, Tatsuhiko Kadowaki

**Affiliations:** 1 Department of Biological Sciences, Xi’an Jiaotong-Liverpool University, Jiangsu Province 215123, China; 2 Department of Physiological Sciences, SOKENDAI (the Graduate University for Advanced Studies), Okazaki, 444-8585, Japan; 3 Division of Cell Signaling, Okazaki Institute for Integrative Bioscience, National Institutes of Natural Sciences, Okazaki, 444-8787, Japan; 4 Department of Entomology, South China Agricultural University, Guangzhou, Guangdong Province 510642, China

**Keywords:** Evolutionary plasticity, fire ant, honey bee, HsTRPA channel, sensor for noxious stimuli

## Abstract

*Solenopsis invicta*, the red imported fire ant, represents one of the most devastating invasive species. To understand their sensory physiology, we identified and characterized their Hymenoptera-specific (Hs) TRPA channel, SiHsTRPA. Consistent with the sensory functions of SiHsTRPA, it is activated by heat, an electrophile, and an insect repellent. Nevertheless, SiHsTRPA does not respond to most of the honey bee ortholog (AmHsTRPA)-activating compounds. The jewel wasp ortholog (NvHsTRPA) is activated by these compounds even though it outgroups both AmHsTRPA and SiHsTRPA. Characterization of AmHsTRPA/SiHsTRPA chimeric channels revealed that the amino acids in the N terminus, as well as ankyrin repeat 2 (AR2) of AmHsTRPA, are essential for the response to camphor. Furthermore, amino acids in ARs 3 and 5–7 were specifically required for the response to diallyl disulfide. Thus, amino acid substitutions in the corresponding domains of SiHsTRPA during evolution would be responsible for the loss of chemical sensitivity. SiHsTRPA-activating compounds repel red imported fire ants, suggesting that SiHsTRPA functions as a sensor for noxious compounds. SiHsTRPA represents an example of the species-specific modulation of orthologous TRPA channel properties by amino acid substitutions in multiple domains, and SiHsTRPA-activating compounds could be used to develop a method for controlling red imported fire ants.

## Visual Abstract

**Figure:**
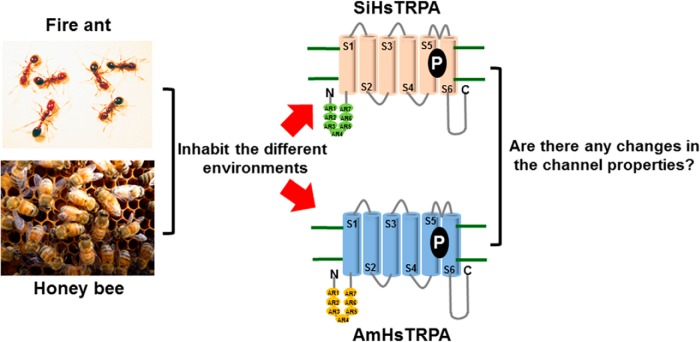

## Significance Statement

TRP channels have crucial roles in the perception of various sensory stimuli by functioning as signal integrators. They have rapidly evolved at multiple levels, and hence the chemical and temperature activation profiles of orthologous TRP channels can differ even between closely related species. Two major questions remain to be answered: what are the physiological consequences of such changes in channel properties, and have these changes been driven by adaptive evolution? The comparison between HsTRPA (SiHsTRPA) of the red imported fire ant and the honey bee ortholog, AmHsTRPA, reported here gives insights into the above questions. Furthermore, SiHsTRPA may serve as a new molecular target in strategies to control the fire ant, a major invasive alien species.

## Introduction

Transient receptor potential (TRP) channels are different from most other cation channels, as they display diverse cation selectivity and activation mechanisms (Venkatachalam and Montell, 2007; Julius, 2013). TRP channels function in various sensory perception pathways such as vision, thermosensation, olfaction, hearing, taste sensation, and mechanosensation (Damann et al., 2008). Individual cells use TRP channels to detect local changes in temperature, osmolarity, and fluid flow in their environment (Venkatachalam and Montell, 2007; Nilius and Owsianik, 2011). TRP channels are evolutionarily plastic and dynamic at multiple levels: gene gain/loss, amino acid substitutions, transcription, and pre-mRNA splicing (Kadowaki, 2015). Thus, TRP channels have undergone more rapid evolution than several conserved cation channels, such as K^+^ channels (Kadowaki, 2015; Saito and Tominaga, 2015).

The metazoan TRP superfamily is classified into eight subfamilies—TRPA, TRPC, TRPM, TRPML, TRPN, TRPP, TRPV, and TRPVL—according to a phylogenetic tree based on the amino acid sequences of six transmembrane segments (Peng et al., 2015a). Among them, the TRPA subfamily contains ankyrin repeats (ARs) in the N terminus. ARs are 33-residue motifs consisting of pairs of antiparallel α-helices connected by β-hairpin motifs and have important roles in the chemical and temperature reactivity of TRPA1 channels (Hinman et al., 2006; Macpherson et al., 2007; Cordero-Morales et al., 2011; Jabba et al., 2014). Among TRPA subfamily members, TRPA1 channels have been best characterized; however, other insect- and crustacean-specific TRPA channels, Painless (Pain), Pyrexia (Pyx), Waterwitch (Wtrw), and HsTRPA, have also been characterized (Tracey et al., 2003; Lee et al., 2005; Liu et al., 2007; Sokabe et al., 2008; Matsuura et al., 2009; Sun et al., 2009; Kohno et al., 2010; Wolfgang et al., 2013). All channels except Wtrw were shown to be heat sensitive, and honey bee HsTRPA (AmHsTRPA) is also activated by electrophiles and camphor (Kohno et al., 2010). Because the ancient nocisensor, TRPA1, is absent in the genome-sequenced Hymenoptera, it was proposed that AmHsTRPA was generated by duplication of honey bee Wtrw and has undergone neofunctionalization to gain heat and chemical sensitivity to complement the loss of TRPA1 function in honey bees (Kohno et al., 2010). Heat activation of jewel wasp (*Nasonia vitripennis*) HsTRPA (NvHsTRPA) was also briefly reported (Matsuura et al., 2009). Evolutionary plasticity was also well documented within TRPA1 channels (Kadowaki, 2015; Saito and Tominaga, 2015). Although gene gain/loss events in other TRPA subfamily members were previously reported (Peng et al., 2015a), plasticity by amino acid substitutions has not yet been shown with any member.

The red imported fire ant (RIFA), *Solenopsis invicta*, is one of the major invasive alien species in the United States, China, Australia, and other regions of the world. The invasion of *S. invicta* not only represents threats to the local ecosystem (Morrison, 2002; Wilder et al., 2013), but also affects agricultural production (Wu et al., 2014). In addition, *S. invicta* causes public health concerns because of the potential serious clinical symptoms of stings (Fernandez-Melendez et al., 2007). Owing to the variety of negative impacts of invasive RIFA (Vinson, 2013), a number of methods to manage RIFAs have been developed, and baits and conventional contact insecticides such as chlorpyrifos and pyrethroids have been widely used. However, the use of these chemicals often creates public health and environmental problems. To avoid these issues, it is important to develop new strategies to control RIFAs. Therefore, identifying safe and natural insecticides/repellents has gotten a lot of attention, and several reports have shown that some plant-derived compounds, particularly from essential oils, effectively repel and/or kill RIFAs (Wheeler et al., 2003; Appel et al., 2004; Chen et al., 2008; Kafle and Shih, 2013; Zhang et al., 2014). However, the mechanisms of how these compounds repel RIFAs have not yet been revealed.

To investigate the functions and evolution of insect and crustacean-specific TRPA subfamily members in insects other than the fruit fly, *Drosophila melanogaster*, and identify potential compounds to repel RIFAs, we identified *S. invicta* HsTRPA (SiHsTRPA) and characterized its physiological functions in comparison with AmHsTRPA. Ants represent a major component of most terrestrial ecosystems, and many species show an enormous diversity in life history traits, ecological and behavioral adaptations, and social organization. Sensory systems of ants must play important roles for the above habitats and behaviors; nevertheless, these systems still remain poorly understood. Characterization of SiHsTRPA should give insight into the roles of TRP channels in the ant sensory system. Additionally, we discuss the evolutionary plasticity of HsTRPA by comparing the properties of SiHsTRPA and AmHsTRPA channels.

## Materials and Methods

### 5′ and 3′ RACE of *SiHsTRPA*

To identify *SiHsTRPA*, we blast searched for *S. invicta* genomic scaffolds (Sinv_1.0_Scaffold_Assembly) and the predicted transcripts (Sinv_OGSv2.2.3_cds) using AmHsTRPA sequence (accession no. LC322996) as a query (Wurm et al., 2011; Elsik et al., 2016). Two sequences similar to AmHsTRPA corresponded to *SiHsTRPA* and *Siwtrw.* To identify 5′ and 3′ ends of *SiHsTRPA*, *S. invicta* total RNA and two primers, 5′-GCGGATAATCCTTCGCTGCATAAGTGC-3′ (for the first PCR) and 5′-AATGGATAGCGGATCGCGTTTGTGACG-3′ (for the second PCR) were used for 5′ RACE with 5′-Full RACE Kit (Takara). 3′ RACE was conducted as above using two primers, 5′-CCCTCGTCTGCGAATGACGACATCAAT-3′ (for the first PCR) and 5′-GCGATCACGCCGTTTGGCTGAAACATC-3′ (for the second PCR) with SMARTer RACE cDNA Amplification Kit (Clontech Laboratories).

### Construction of SiHsTRPA-expressing vector for mammalian cells

We isolated full-length *SiHsTRPA* cDNA by RT-PCR using *S. invicta* total RNA and the following two primers; 5′-TGAATTCACC**ATG**TGTGAGAATGATAAAGATTCGTTAGG-3′ and 5′-AATTTGCGGCCGCCTAATGAGGTATTTTCTTCTTTCGCCAATGT-3′. The PCR product was digested with EcoRI and NotI, and then cloned in pAc5.1/V5-His B vector (Thermo Scientific) in which *D. melanogaster actin 5C* promoter was replaced with CMV promoter. The SiHsTRPA protein expressed by this construct was tagged with a V5-epitope at the C terminus, and this was used for verifying the expression and the cellular localization in HEK 293 cells (ATCC PTA-5077, RRID:CVCL_6911) by Western blot and immunofluorescence with rabbit anti-V5-epitope antibody (Sigma-Aldrich V8137, RRID:AB_26188), respectively. The staining pattern of V5-epitope-tagged SiHsTRPA channel was compared with FITC-WGA, which specifically labels the plasma membrane (Chazotte, 2011). A construct expressing untagged SiHsTRPA protein was then prepared using the above DNA construct as a template, the above primer with initiation codon, and the primer 5′-AATTTGCGGCCGCCCTATAATGAGGTATTTTCTTCTTTCGCCAA-3′. This DNA construct was used for all of the experiments described in the text.

### Construction of SiHsTRPA and AmHsTRPA mutants as well as AmHsTRPA/SiHsTRPA chimeric channels

N-terminal deletion (amino acid 21–98) mutant of AmHsTRPA was constructed by overlap extension PCR (Lee et al., 2010). Two PCR products were first prepared using the following primers; 5′-AATTAACCCTCACTAAAGGG-3′ (T3) and 5′-ATCCGTGTAAAGTTGCAAGCTAGCCTT-3′ (P2; upstream fragment) and 5′-TTGCAACTTTACACGGATGAAGACAGA-3′ (P3) and 5′-CGATCAACTGCGTTGACATTG-3′ (P4; downstream fragment) and *AmHsTRPA* expression vector (Kohno et al., 2010) as a template. The second PCR was performed by mixing above two PCR products and using T3 and P4 as the primers, and then the resulting PCR product was digested with NotI and XbaI followed by replacing the corresponding fragment in the *AmHsTRPA* expression vector. AmHsTRPA N-terminal insertion (amino acid 1–98) to SiHsTRPA was also constructed as above. The upstream fragment was prepared using primers, 5′-TTTAAGAATTCACCATGGACGATAAACAGATA-3′ (P5) and 5′-CTCACACATCAGGCAGGAACGACGATT-3′ (P6) and the *AmHsTRPA* expression vector as a template. The downstream fragment was prepared using primers, 5′-TCCTGCCTGATGTGTGAGAATGATAAA-3′ (P7) and 5′-TCTCTCGCTTTTGCACGCGTT-3′ (P8) and the *SiHsTRPA* expression vector as a template. The second PCR was performed by mixing above two PCR products and using P5 and P8 as the primers, and then the resulting PCR product was digested with EcoRI and SphI followed by replacing the corresponding fragment in the *SiHsTRPA* expression vector.

All of the AmHsTRPA/SiHsTRPA chimeric channels were constructed by overlap extension second PCR of the two or three first PCR amplicons. Both upstream and downstream fragments were PCR amplified using the *AmHsTRPA* expression vector as a template and middle fragment was PCR amplified using the *SiHsTRPA* expression vector as a template. The N terminus swapped AmHsTRPA chimera was constructed using three amplicons; the upstream fragment prepared by T3 and 5′-CGAATCTTTGGTCAAGAAATTTCTGTC-3′ (P9) primers, the downstream fragment prepared by 5′-GAGAGACTAAGAAATATAGCGTACATG-3′ (P10) and P4 primers, and the middle fragment prepared by 5′-TTCTTGACCAAAGATTCGTTAGGAGAC-3′ (P11) and 5′-TATATTTCTTAGTCTCTCGCTTTTGCA-3′ (P12). The AR1-swapped AmHsTRPA was constructed using three amplicons; the upstream fragment prepared by T3 and 5′-GTTCCGTAGTCGTTCTCCTGCCGGTAT-3′ (P13) primers, the downstream fragment prepared by 5′-GAACCGCGAACGGGCATGAACGCGATC-3′ (P14) and P4 primers, and the middle fragment prepared by 5′-GGAGAACGACTACGGAACGTCGCTTAC-3′ (P15) and 5′-CATGCCCGTTCGCGGTTCGACGTAGTT-3′ (P16). The AR2-swapped AmHsTRPA was constructed using three amplicons; the upstream fragment prepared by T3 and 5′-CACGCCGGTGCTAGGCTCCACGTAATC-3′ (P17) primers, the downstream fragment prepared by 5′-GAGAGCCTCATCAATCATTACACGCCT-3′ (P18) and P4 primers, and the middle fragment prepared by 5′-GAGCCTAGCACCGGCGTGAACGCTATC-3′ (P19) and 5′-ATGATTGATGAGGCTCTCATAATTTAC-3′ (P20). The AR3-swapped AmHsTRPA was constructed using three amplicons; the upstream fragment prepared by T3 and 5′-GTAATGATTGATGAGGCTCTCATAATT-3′ (P21) primers, the downstream fragment prepared by 5′-TACGTCTGTCAGGAGGTGGCCGAACCA-3′ (P22) and P4 primers, and the middle fragment prepared by 5′-AATCCGATCACTCGTTACACACCGCTT-3′ (P23) and 5′-CACCTCCTGACAGACGTAATTGAGCTT-3′ (P24). The second PCR for above chimeras was performed using T3 and P4 as the primers, and the amplicon was processed as above for N-terminal-deletion AmHsTRPA mutant. The AR3-5 swapped AmHsTRPA was constructed using three amplicons; the upstream fragment prepared by T3 and 5′-GTAACGAGTGATCGGATTCCGGTAATT-3′ (P25) primers, the downstream fragment prepared by 5′-GTCGACAGGGCACACAGAACCCCACTT-3′ (P26) and 5′-GCCTCGTCGTGGTTGTAACA-3′ (P27) primers, and the middle fragment prepared by 5′-AATCCGATCACTCGTTACACACCGCTT-3′ (P28) and 5′-TCTGTGTGCCCTGTCGACGGCGTTCAC-3′ (P29). The second PCR was performed using T3 and P27 as the primers, and then the amplicon was digested with NotI and EcoRI followed by replacing the corresponding fragment in the *AmHsTRPA* expression vector. The AR5-7 swapped AmHsTRPA was constructed as above except using the following three amplicons; the upstream fragment prepared by T3 and 5′-CTGGCCCAGATGGTTTTTCTCGACGAC-3′ (P30) primers, the downstream fragment prepared by 5′-GTCGACAGGGCACACAGAACCCCACTT-3′ (P31) and P27 primers, and the middle fragment prepared by 5′-AAAAACCATCTGGGCCAGACGCCGTTG-3′ (P32) and 5′-AAGTCCCGCGCGCGTGGTCGCACTGAT-3′ (P33). The boundary between ARs and transmembrane segments swapped AmHsTRPA was constructed using three amplicons; the upstream fragment prepared by 5′-TCATGTGGTCGATCGTCACT-3′ (P34) and 5′-GCCGGTGCGGGTGGTCGCACTGACGTCCGCACCGGCCCAAATTA-3′ (P35) primers, the downstream fragment prepared by 5′-CAGGAGCACCGTAAAGATTTGCTCTCC-3′ (P36) and P27 primers, and the middle fragment prepared by 5′-GCGACCACCCGCACCGGCTTGTCTGCT-3′ (P37) and 5′-ATCTTTACGGTGCTCCTGTACGAAAGT-3′ (P38). The second PCR was performed using P34 and P27 as the primers, and then the amplicon was digested with XbaI and EcoRI followed by replacing the corresponding fragment in the *AmHsTRPA* expression vector. The selectivity filter swapped AmHsTRPA was constructed using three amplicons; the upstream fragment prepared by 5′-CCGTTGGTAATGGCTTTCCT-3′ (P39) and 5′-CAATTCACCGGCCATCATTGCCAATAC-3′ (P40) primers, the downstream fragment prepared by 5′-CTCTTCACGCTGTTCATCGTGTTTGTT-3′ (P41) and 5′-AAGGCTCTCAAGGGCATCGGT-3′ (P42) primers, and the middle fragment prepared by 5′-ATGATGGCCGGTGAATTGGATTTCGAA-3′ (P43) and 5′-GATGAACAGCGTGAAGAGGATCTGCGA-3′ (P44). The second PCR was performed using P39 and P42 as the primers, and then the amplicon was digested with BamHI and EcoRI followed by replacing the corresponding fragment in the *AmHsTRPA* expression vector. The C terminus swapped AmHsTRPA was constructed using two amplicons; the upstream fragment prepared by P39 and 5′-CGCAGACGACGGTATATTTTTCTGTGC-3′ (P45) primers and the downstream fragment prepared by 5′-AATATACCGTCGTCTGCGAATGACGAC-3′ (P46) and P42 primers. The second PCR followed by the restriction enzyme digestion were the same as above.

### RT-PCR and qRT-PCR

Total RNA was isolated from the abdomen, antennae, heads, legs, and thoraxes of 20 fire ants using Trizol reagent (Thermo Fisher Scientific). Total RNA was reverse transcribed using random primer and ReverTra Ace reverse transcriptase (Toyobo). The RT products were then used for PCR with the following primers; 5′-TAACTGGCGTGATGTGGGAC-3′ and 5′-CATGCACGCAGTATTCAGGC-3′ (for *S. invicta 18S rRNA*), 5′-AGGTCTGAGGAAACACGCAG-3′ and 5′-TCGGAAGGCATACGGGATTG-3′ (for *SiHsTRPA*). The PCR was repeated for 25 and 35 cycles for *18S rRNA* and *SiHsTRPA*, respectively. The resulting PCR products were sequenced to verify their identities. The same primer sets and SYBR Green were used for qRT-PCR, and total RNA samples were prepared from three batches of collected tissues.

### Ca^2+^-imaging method (HEK293 cells)

For Ca^2+^-imaging, 1 µg SiHsTRPA expression vector and 0.1 µg pCMV-DsRed expression vector were transfected to HEK293 cells in OPTI-MEM medium (Thermo Fisher Scientific) using Lipofectamine Plus reagents (Thermo Fisher Scientific). After incubating for 3–4 h at 37°C, cells were reseeded on cover glasses and further incubated at 33°C. The cells were used for the experiments 20–40 h after transfection. Transfected HEK293 cells on a cover glass were incubated in culture medium containing 5 µM fura-2 am (Thermo Fisher Scientific) at 33°C for 1–2 h. The cover glass was washed, and fura-2 fluorescence was measured in a standard bath solution containing (in mm) 140 NaCl, 5 KCl, 2 MgCl_2_, 2 CaCl_2_, 10 Hepes, and 10 glucose, pH 7.4 adjusted with NaOH. A cover glass was mounted in a chamber (RC-26G, Warner Instruments) connected to a gravity flow system to deliver hot or cold bath solution for temperature stimulations and bath solution containing the tested compounds. The concentration of each compound was 1 mm except for carvacrol, geranylacetone, nerol (0.5 mm), menthol (3 mm), camphor (5 mm), saturated fatty acid (0.2 mm), and creosote (0.1%). The emitted fluorescence (510 nm) by 340 and 380 nm were measured by CCD camera (CoolSNAP ES, Roper Scientific Photometrics), and the Fura-2 ratios (340/380 nm) were acquired and recorded every 3 s by IPlab software (Scanalytics). Changes of Fura-2 ratio over time were analyzed by ImageJ. Each recording was repeated twice except for stimulation of SiHsTRPA by heat or β-citronellol and stimulation of AmHsTRPA by octanoic acid (three times each). To assess activation of the channel by the specific stimulation, we calculated the fold change of Fura-2 ratio (the maximum Fura-2 ratio recorded after the stimulation divided by Fura-2 ratio just before the stimulation) in the individual DsRed-positive (with the channel expression) and DsRed-negative (without the channel expression) cells, and then statistically compared the values.

### Genetics

*UAS-SiHsTRPA* fruit fly was generated by integrating the transgene to 68A4 by PhiC31 integrase-mediated recombination event (Bischof et al., 2007). The transgene was driven by *Gr33a-Gal4* (Moon et al., 2009). The above transgene was also recombined with *20XUAS-IVS-GCaMP6m* (Chen et al., 2013) on the third chromosome, and then their expression was driven by *Gr64f-Gal4* (Jiao et al., 2008) with two copies for each *UAS* and *Gal4* transgene.

### Assay for thermotactic behavior of *D. melanogaster*

To assay the temperature preference of fruit flies, a temperature gradient of 14–37°C with a slope of 0.94°C/cm was produced in an aluminum block (27 long × 15 wide × 2.5 cm high) as previously reported (Sayeed and Benzer, 1996). Thermometers were embedded in the block every 2.4 cm, and the gradient was established using a cold circulating water chamber and a hot probe at each end. The aluminum block was covered with moist paper to maintain a uniform relative humidity along the gradient. This paper was divided into 20 observation fields with a black pencil for recording the distribution of fruit flies. A glass plate with three separate lanes was placed 5 mm above the block, creating suitable corridors for fruit flies to migrate. Forty to fifty fruit flies per lane were placed in the middle of the testing arena around 25°C between the aluminum block and the glass plate, allowed to migrate for 3 h, and photographed every 10 min with a digital camera. Fruit flies moved very rapidly in the entire area when they were first introduced into the apparatus; however, they gradually ceased rapid movement in the apparatus. We then counted the number of fruit flies in each observation field and divided by the total number of fruit flies in the entire area every 10 min between 1.5 and 2.5 h after the introduction. The average value of six recordings was calculated for each experimental group. Before introducing fruit flies into the temperature gradient apparatus, they were placed in plastic vials and cooled on ice until they stopped moving. All experiments were performed in a room where the temperature was kept constant at 25°C.

### Assay for thermal nociception behavior of *D. melanogaster* larvae

To test the thermal nociception behavior of *D. melanogaster* larvae, the actively wandering third instar larvae were collected from vials and transferred to 35-mm plastic Petri dishes with water to support exhibiting the full rolling response. Larvae were touched laterally in abdominal segments (A4–A6) with the hot probe. In all experiments, 20–40 larvae were tested per group in four replicates. The behavioral responses were recorded and analyzed as described (Tracey et al., 2003; Neely et al., 2011; Zhong et al., 2012). To calculate the median of response latency of each genotype, the latency of larvae responding over 10 s was considered as 10, although some of the larvae never responded to touching by the hot probe.

### Proboscis extension response assay of *D. melanogaster*

The proboscis extension response (PER) assay was performed basically as described in (Peng et al., 2015b). Two- to three-day-old fruit flies were starved overnight on wet Kim Wipe, briefly anaesthetized on ice, and affixed to a tooth pick. Fruit flies recovered in a humidified chamber for at least 1 h at room temperature before testing. Each subject was checked for intact PER before beginning the experiments. Flies that did not show the reflex were discarded. During the PER assay, the fruit fly was first satiated with water, and 100 mm sucrose with 10% DMSO containing 0 or 0.6 mm decanoic acid (WAKO) to activate SiHsTRPA was touched to the forelegs with a pipette tip. If the proboscis was extended and contact with the sucrose solution was maintained for 3 s, the response was scored as 1. If the contact of proboscis was brief, a 0.5 was awarded. If the proboscis failed to contact the solution within 5 s of offering, a 0 was awarded. Each fruit fly was offered compounds seven times per experiment, and between offerings water was given to satiation. Because decanoic acid was accepted on first offering (perhaps because the first activation of aversive taste neurons by SiHsTRPA was not sufficient to repress proboscis extension induced by activation of sugar taste neurons with 100 mm sucrose), PER frequency was calculated for the second through to the seventh offerings (sum of 6 scores per fruit fly divided by 6). Three groups of 7–8 flies per genotype were tested. For PER induction by decanoic acid, the forelegs of fruit flies prepared as above were touched with the 0.6-mm solution in 10% DMSO, and then the extension of proboscis was scored followed by checking the intact PER by sucrose.

### GCaMP6 imaging in *D. melanogaster*

For the GCaMP6 imaging, we first cut the female foreleg between the femur and the tibia and dipped the first three tarsal segments and the tibia in silicone oil. We then placed the severed legs laterally on double-sided tape pasted to a glass slide. Only the fourth and fifth tarsal segments were exposed and put under 5 µL of 10% DMSO by covering the tibia and the first three tarsal segments of the leg with 2% agarose (Miyamoto et al., 2013). Three to four images were obtained first (0 s), and then time-lapse recording (every 0.5 s for 30 s) by a CCD camera (RETIGA 2000-RV, Roper Scientific Photometrics) was started by adding 5 µL of 1.2 mm decanoic acid in 10% DMSO. Data were acquired and analyzed by Image-Pro Plus software (Media Cybernetics).

### Repellent assay with *S. invicta* in the laboratory

Two filter papers were soaked with either 10% DMSO or repellent in 10% DMSO, and then briefly dried up on a paper towel. We joined two filter papers side by side and introduced 20 *S. invicta* workers (briefly anaesthetized on ice) at the boundary at the beginning of the assay (0 min), and they were allowed to move for 40 min in the testing arena (9-cm-diameter circle). The number of workers in the semicircle area with either DMSO or repellent was counted every 5 min for 10–40 min, and the avoidance index was calculated by the sum of seven recordings. Two filter papers soaked with 10% DMSO were used as a control assay. The experiments were repeated three times for each concentration of β-caryophyllene, β-citronellal, β-citronellol, octanoic acid, and decanoic acid shown in Fig. 8.

### Statistical analysis

The results of Fig. 2*B*,*C* were analyzed by a two-tailed Dunnett test and two-tailed Steel test, respectively. The results of Fig. 8*B–E* were analyzed by two-tailed Dunnett test. The results of Fig. 9*A*,*C*,*D* were analyzed by two-tailed *t* test. The results of calcium imaging in Figs. 2–7 were analyzed by one-tailed Welch’s *t* test.

## Results

### Identification of SiHsTRPA and its mRNA expression profile in *S. invicta*

Using *AmHsTRPA* sequence as a query, we searched for an ortholog in the annotated *S. invicta* gene set and genomic scaffolds and found two potential sequences. They represent *S. invicta HsTRPA* (*SiHsTRPA*) and *Wtrw*, respectively. Similar to other Hymenopteran insects, these two genes are located in the same genomic scaffold (04103) in tandem and are 3.7 kb apart. We obtained the full-length *SiHsTRPA* cDNA by using primers designed from the sequences of both 5′ and 3′ RACE products (accession no. LC188017). We did not identify any isoforms and obtained a single cDNA. SiHsTRPA contains seven ARs at the N terminus followed by the ion-transport domain with six transmembrane segments. This structure is common between HsTRPA channels including AmHsTRPA (Kohno et al., 2010).

**Figure 1.**
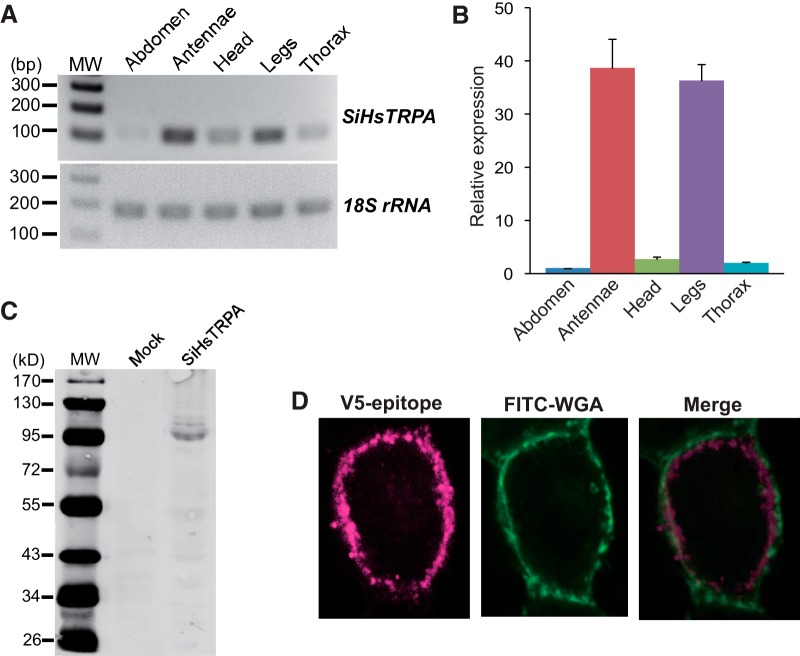
Expression profile of *SiHsTRPA* mRNA and the protein expression in HEK293 cells. ***A***, Detection of *SiHsTRPA* mRNA and *S. invicta* 18S rRNA in the abdomen, antennae, head, legs, and thorax by RT-PCR. The position of 100–300-bp DNA molecular weight marker (MW) is shown at the left. ***B***, Relative expression levels of *SiHsTRPA* mRNA in the abdomen, antennae, head, legs, and thorax measured by qRT-PCR. *S. invicta* 18S rRNA was used as the internal standard, and the level in the abdomen was set as 1. ***C***, Proteins expressed in HEK293 cells transfected with empty vector (Mock) and SiHsTRPA-expressing construct were analyzed by Western blot. The size (in kilodaltons) of protein molecular weight marker (MW) is at the left. ***D***, Localizations of plasma membrane-bound FITC-WGA and SiHsTRPA tagged with V5-epitope in the transfected HEK293 cells by immunofluorescence. The merged image is also shown.

We examined the expression of *SiHsTRPA* mRNA in the abdomen, antennae, head, legs, and thorax of *S. invicta* by using reverse-transcription PCR (RT-PCR). 18S rRNA was equally present in all samples; however, *SiHsTRPA* mRNA was abundant in the antennae and legs compared with other tissues (Fig. 1*A*). These results were also confirmed by quantitative RT-PCR (qRT-PCR; Fig. 1*B*).

### SiHsTRPA protein expression in HEK293 cells

Before characterizing the channel properties of SiHsTRPA, we first characterized the protein expression and cellular localization of V5-epitope-tagged SiHsTRPA in HEK293 cells. Specifically, we detected the protein with the expected molecular weight, 104 kDa, by Western blot analysis (Fig. 1*C*). We then examined the cellular localization of the epitope-tagged SiHsTRPA by double staining of the expressing cells with FITC-WGA (to label the plasma membrane) and anti-V5-epitope antibody. As shown in Fig. 1*D*, most of the protein was present slightly beneath the plasma membrane labeled by FITC-WGA; however, a fraction of the protein was localized at the plasma membrane.

### Heat-evoked activation of SiHsTRPA

**Figure 2.**
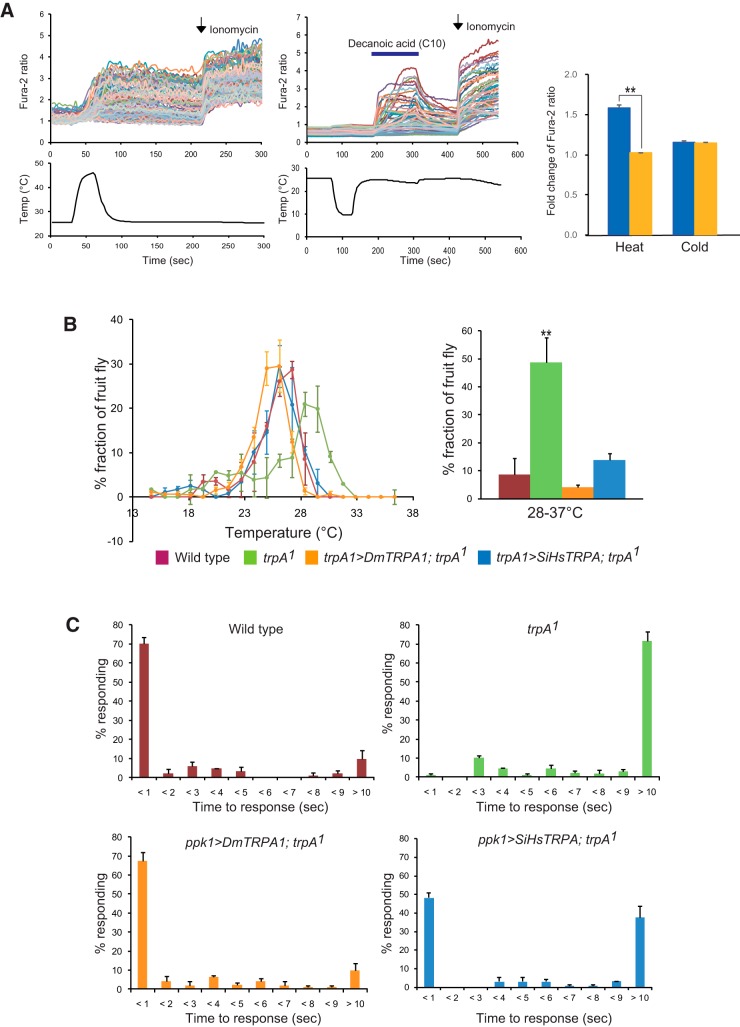
Heat activation of SiHsTRPA and the effects of introduction on the temperature-related phenotypes of *D. melanogaster trpA^1^* mutant. ***A***, The upper traces indicate the changes of intracellular calcium level in SiHsTRPA-expressing cells by high (left) and low (right) temperatures. The lower traces show the changes of bath temperature by time (sec, second). Each line represents the intracellular calcium level in the individual cell measured by calcium imaging. Decanoic acid (C10) was added at 180–300 s (blue bar) after cold stimulation to confirm that the channels expressed in the cells were functional. The arrows show the time points when 5 μm ionomycin was added. The mean value of fold change of Fura-2 ratio with error bar (± SEM) is shown for the heat- or cold stimulated DsRed-positive (blue) and DsRed-negative (yellow) cells. The total numbers of cells analyzed were 79–228. Significant difference is shown by (**, *P* < 0.001, one-tailed Welch’s *t* test). ***B***, The distribution of wild-type, *trpA1^1^*, and *trpA1^1^* expressing either *DmTRPA1* (*trpA1 > DmTRPA1; trpA1^1^*) or *SiHsTRPA* (*trpA1 > SiHsTRPA; trpA1^1^*) under *trpA1-Gal4* was recorded along a thermal gradient (14–37°C). The recording was repeated three times for each group. The mean value with error bar (± SEM; *n* = 3) is shown for each temperature section (left). The percentage of fruit flies in the area of 28–37°C (wild type: 8.6 ± 5.9%; *trpA1^1^*: 48.8 ± 8.9%; *trpA1^1^*expressing *DmTRPA1*: 4.1 ± 0.9%; *trpA1^1^*expressing *SiHsTRPA*: 13.7 ± 2.6%) of the thermal gradient. Only *trpA1^1^* (**) is significantly different from wild type (two-tailed Dunnett test, *P* < 0.003; right). ***C***, Wild type (*n* = 92), *trpA1^1^* (*n* = 90), and *trpA1^1^* expressing either *DmTRPA1* (*ppk1 > DmTRPA1; trpA1^1^*, *n* = 83) or *SiHsTRPA* (*ppk1 > SiHsTRPA; trpA1^1^*, *n* = 125) under *ppk1.9-Gal4* were tested for their response to high temperature using a 46°C probe. The percentage of responding larvae for each genotype is shown at each second within 9 s and >10 s (mean value ± SEM). *P*-values (two-tailed Steel test) for wild type and *trpA1^1^* expressing either *DmTRPA1* or *SiHsTRPA* compared to *trpA1^1^*are <0.023, <0.023, and <0.038, respectively. *P*-value (two-tailed Steel test) for *trpA1^1^* expressing *SiHsTRPA* is <0.12 compared with wild type.

We measured the relative changes in intracellular calcium levels in HEK293 cells expressing SiHsTRPA by temperature fluctuations using a Fura-2 calcium imaging technique. Activation of the channel results in increasing the intracellular calcium levels by an influx of extracellular calcium. As shown in Fig. 2*A*, elevation but not reduction in temperature increased the relative intracellular calcium levels of cells expressing SiHsTRPA. Decanoic acid (C10) was used as a positive control to verify that SiHsTRPA expressed in the cell was functional. These results suggest that SiHsTRPA is a heat-activated TRPA channel.

### SiHsTRPA rescues the impaired thermotactic and thermal nociception behaviors of *D. melanogaster trpA1* mutant

The above results revealed that SiHsTRPA is activated by heat. We, therefore, tested the thermotactic behaviors of wild-type, *trpA1^1^* (Kwon et al., 2008), and *trpA1^1^* adults expressing *DmTRPA1* or *SiHsTRPA* under *trpA1-Gal4* (Rosenzweig et al., 2005; Kim et al., 2010) by using an aluminum block with a temperature gradient of 14–37°C (Sayeed and Benzer, 1996). This thermotactic behavior of adult *D. melanogaster* would depend on not only DmTRPA1 but also other thermosensors such as Pyx and Gr28b (Lee et al., 2005; Ni et al., 2013). Although wild-type adult fruit flies preferred temperatures ∼25–26°C and avoided temperatures >28°C (Fig. 2*B*), *trpA1^1^* adults preferred high temperatures, and more flies were found in the test zones where the temperatures were >28°C (Fig. 2*B*; Hamada et al., 2008). However, this behavioral defect was rescued by expressing either *DmTRPA1* or *SiHsTRPA* (Fig. 2*B*).

We also tested the thermal nociception behaviors of the wild-type, *trpA1^1^*, and *trpA1^1^* larvae expressing either *DmTRPA1* or *SiHsTRPA* in the class IV multidendritic (mdIV) neurons under *ppk1.9-Gal4* (Ainsley et al., 2008). For this, we characterized the stereotyped nocifensive escape locomotion behavior on touch, with a 46°C probe (Tracey et al., 2003; Neely et al., 2011; Zhong et al., 2012). The mdIV neurons function as the polymodal nociceptors of larvae. Most of the wild-type larvae initiated escape behavior within 1 s as previously reported; however, *trpA1^1^* larvae displayed a significantly delayed response (Fig. 2*C*; Neely et al., 2011; Zhong et al., 2012). In fact, many of the mutant larvae never showed the escape behavior (Fig. 2*C*). Introducing either *DmTRPA1* or *SiHsTRPA* improved the impaired nociceptive behavior of *trpA1^1^* larvae; however, SiHsTRPA appeared to rescue the phenotype to a lesser extent than did DmTRPA1. In fact, >30% of the larvae showed response latency of >10 s (Fig. 2*C*). Nevertheless, the *trpA1^1^* larvae expressing *SiHsTRPA* group did not show a statistically significant difference from the wild type. These results suggest that SiHsTRPA could complement the functions of DmTRPA1 together with Pain and Pyx (Tracey et al., 2003; Neely et al., 2011; Zhong et al., 2012) for thermal nociceptive behavior in fruit fly larvae.

### SiHsTRPA and AmHsTRPA show different chemical responses

**Figure 3.**
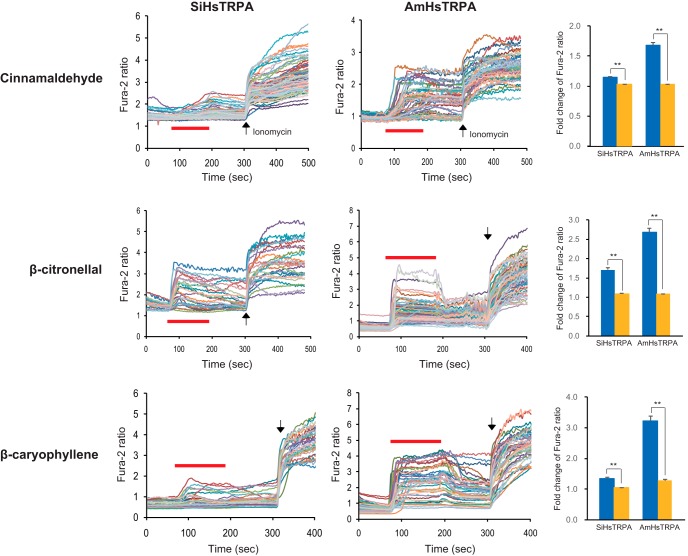
Activation of both SiHsTRPA and AmHsTRPA by three plant-derived compounds. Activation of both SiHsTRPA and AmHsTRPA by cinnamaldehyde, β-citronellal, and β-caryophyllene was analyzed by calcium imaging. Red bars show the period when each compound was added, and then washed off. Arrows show the time points when ionomycin was added. The concentration of each compound tested was 1 mm. The mean value of fold change of Fura-2 ratio with error bar (± SEM) is shown for the compound-stimulated DsRed-positive (blue) and DsRed-negative (yellow) cells expressing SiHsTRPA or AmHsTRPA. The total numbers of cells analyzed were 70–206. **, Significant difference by one-tailed Welch’s *t* test (*P* < 0.001).

**Figure 4.**
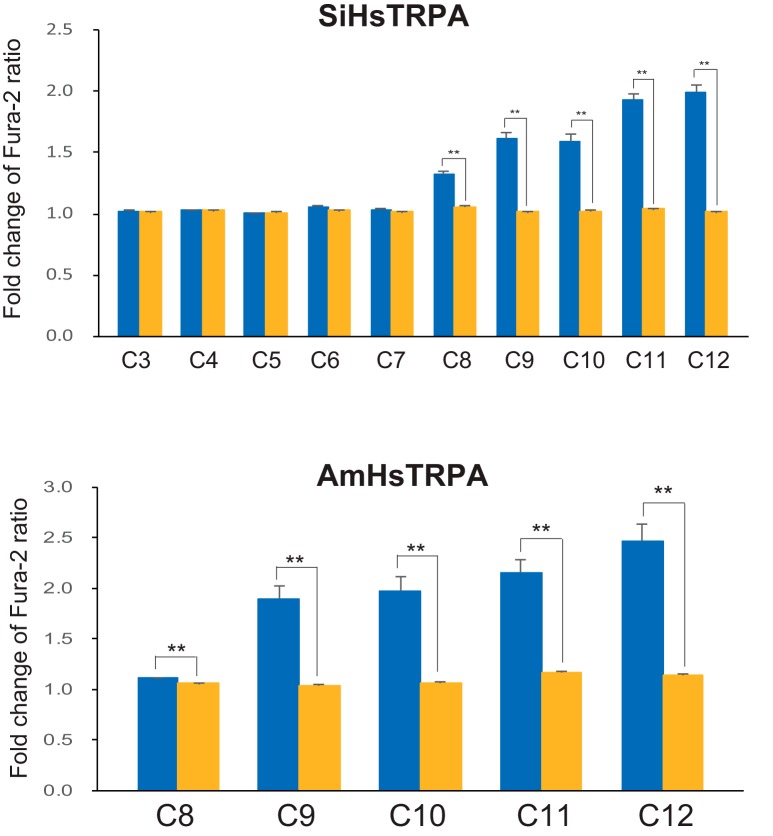
Activation of SiHsTRPA and AmHsTRPA by saturated medium-chain fatty acids. Activation of SiHsTRPA and AmHsTRPA by saturated fatty acids was analyzed by calcium imaging. The mean value of fold change of Fura-2 ratio with error bar (± SEM) is shown for the fatty acid (propionic acid, C3; butyric acid, C4; valeric acid, C5; heptanoic acid, C6; hexanoic acid, C7; octanoic acid, C8; nonanoic acid, C9; decanoic acid, C10; undecanoic acid, C11; and lauric acid, C12) stimulated DsRed-positive (blue) and DsRed-negative (yellow) cells. The total numbers of SiHsTRPA- and AmHsTRPA-transfected cells analyzed were 97–212 and 61–157, respectively. **, Significant difference by one-tailed Welch’s *t* test (*P* < 0.001). The traces in extended data Figs. 4-1 and 4-2 indicate the changes of intracellular calcium level in SiHsTRPA- and AmHsTRPA-expressing cells by above fatty acids, respectively.

AmHsTRPA was shown to be activated by electrophilic compounds (allyl isothiocyanate and cinnamaldehyde) and camphor. However, no other AmHsTRPA-activating compounds were reported (Kohno et al., 2010). Because many plant-derived tick repellents were shown to activate the honey bee ectoparasitic mite TRPA1 channels (Peng et al., 2015b; Dong et al., 2016), we tested the effects of these repellents on activation of both SiHsTRPA and AmHsTRPA channels, using a calcium-imaging technique. We found that 19 compounds activated AmHsTRPA while only three compounds activated SiHsTRPA. The list of active and inactive compounds that open SiHsTRPA and AmHsTRPA channels is shown in Table 1. Fig. 3 shows the activation of SiHsTRPA and AmHsTRPA by cinnamaldehyde, β-citronellal, and β-caryophyllene. Cinnamaldehyde is an electrophilic compound (Macpherson et al., 2007), and β-citronellal has been used as insect repellent (Sakulku et al., 2009). β-Caryophyllene was shown to repel *S. invicta* (Wheeler et al., 2003; Kafle and Shih, 2013). We also identified two saturated fatty acids, decanoic acid (C10) and lauric acid (C12), as SiHsTRPA-activating compounds during an initial screen. We then tested the effects of saturated fatty acids with different numbers of carbon on SiHsTRPA (Figs. 4 and extended data Fig. 4-1). We found that saturated fatty acids with C3–C7 (propionic acid, butyric acid, valeric acid, hexanoic acid, and heptanoic acid) were inactive, but those with >C8 (octanoic acid) were able to activate SiHsTRPA. These results demonstrate that a minimum of eight carbons are necessary for a saturated fatty acid to activate SiHsTRPA. Contrary to other compounds, the activation by fatty acids is quite similar between AmHsTRPA and SiHsTRPA (Fig. 4 and extended data Fig. 4-2).

**Table 1.** List of active and inactive compounds to open SiHsTRPA and AmHsTRPA channels

Active compounds to open SiHsTRPA	Inactive compounds to open SiHsTRPA	Active compounds to open AmHsTRPA	Inactive compounds to open AmHsTRPA
Fatty acids (C8, 1.32; C9, 1.619; C10, 1.596; C11, 1.93; C12, 1.991)	Fatty acids, C3-C7	Fatty acids (C8, 1.108; C9, 1.89; C10, 1.973; C11, 2.15; C12, 2.464)	3 mm menthol
Cinnamaldehyde (1.146)	Diallyl disulfide	Cinnamaldehyde (1.678)	0.5 mm carvacrol
β-Citronellal (1.704)	β-Citronellol	Diallyl disulfide (1.216)	α-Terpineol
β-Caryophelene (1.342)	Camphor	β-Citronellal (2.681)	2-Undecanone
	0.5 mm geranylacetone	β-Citronellol (1.634)	Terpinen-4-ol
	Eugenol	β-Caryophelene (3.228)	0.1% creosote
	Nerolidol	Camphor (1.421)	*o*-Methoxyphenol
	0.5 mm nerol	0.5 mm nerol (1.311)	2-Methoxy-4-methylphenol
	Thujone	Thujone (1.432)	*N*,*N*-diethyl-2-phenylacetamide
	Myrtenal	Nerolidol	Methyl salicylate
	β-Cyclocitral	0.5 mm geranylacetone	Verbenone
	2-Dodecanone	Eugenol	Coumarin
	2-Ethyl-1,3-hexanediol	Myrtenal	Thymol
	Methyl jasmonate	β-Cyclocitral	
	3 mm menthol	2-Dodecanone	
	0.5 mm carvacrol	2-Ethyl-1,3-hexanediol	
	α-Terpineol	Methyl jasmonate	
	2-Undecanone	Geraniol	
	Terpinen-4-ol	Borneol	
	Thymol	AITC	
	2-Methoxy-4-methylphenol		
	Geraniol		
	o-Methoxyphenol		
	Coumarin		
	Verbenone		
	0.1% Creosote; methyl salicylate; borneol; *N*,*N*-diethyl-2-phenylacetamide; AITC		

All compounds were tested at 1-mm concentration except carvacrol, creosote, geranylacetone, nerol, and menthol, at the indicated concentrations. The average fold changes of Fura-2 ratio induced by active compounds shown in this report are indicated with parentheses.

10.1523/ENEURO.0327-17.2018.f4-1 Figure 4-1 Activation of SiHsTRPA by saturated medium- but not short-chain fatty acids. Activation of SiHsTRPA by saturated short- and medium-chain fatty acids was analyzed by calcium imaging. Red bars show the period when each fatty acid was added, and then washed off. C10 was also applied after testing two of the fatty acids (C3 and C8) to confirm the expression of functional channel as indicated by blue bars. Arrows show the time points when ionomycin was added. The concentration of each fatty acid was 0.2 mm. Download Figure 4-1, EPS file.

10.1523/ENEURO.0327-17.2018.f4-2 Figure 4-2 Activation of AmHsTRPA by saturated medium-chain fatty acids. Activation of AmHsTRPA by saturated medium-chain fatty acids was analyzed by calcium imaging. Red bars show the period when each fatty acid was added, and then washed off. C10 was also applied after testing C8 as indicated by blue bar. Arrows show the time points when ionomycin was added. The concentration of each fatty acid was 0.2 mm. Download Figure 4-2, EPS file.

**Figure 5.**
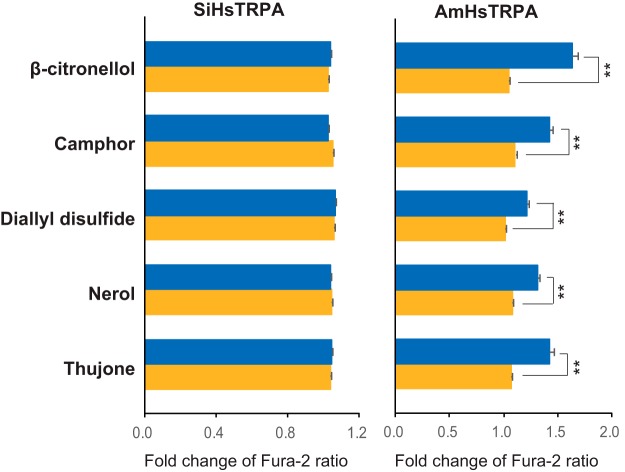
Five plant-derived compounds activate AmHsTRPA but not SiHsTRPA. Responses of SiHsTRPA and AmHsTRPA to β-citronellol, camphor, diallyl disulfide, nerol, and thujone were tested by calcium imaging. The mean value of fold change of Fura-2 ratio with error bar (± SEM) is shown for the compound-stimulated DsRed-positive (blue) and DsRed-negative (yellow) cells. The total numbers of SiHsTRPA- and AmHsTRPA-transfected cells analyzed were 73–153 and 71–103, respectively. **, Significant difference by one-tailed Welch’s *t* test (*P* < 0.001). The traces in extended data Fig. 5-1 indicate the changes of intracellular calcium level in SiHsTRPA- and AmHsTRPA-expressing cells by above five compounds.

As shown in Fig. 5, β-citronellol, camphor, diallyl disulfide, nerol, and thujone activated AmHsTRPA but not SiHsTRPA (extended data Fig. 5-1). In fact, most AmHsTRPA-activating compounds did not activate SiHsTRPA (Table 1). Thus, chemical activation profiles are quite different between these two HsTRPA orthologs of honey bee and RIFA, which belong to the same insect order, Hymenoptera. Intriguingly, β-citronellal with a carbonyl group activated both SiHsTRPA and AmHsTRPA (Fig. 3); however, β-citronellol with a hydroxyl group activated only AmHsTRPA (Fig. 5).

10.1523/ENEURO.0327-17.2018.f5-1 Figure 5-1 Five plant-derived compounds activate AmHsTRPA but not SiHsTRPA. Responses of SiHsTRPA and AmHsTRPA to nerol, thujone, camphor, diallyl disulfide, and β-citronellol were tested by calcium imaging. Red bars show the period when each compound was added, and then washed off. C10 was applied after testing the compounds to confirm the expression of functional SiHsTRPA as indicated by blue bars. Arrows show the time points when ionomycin was added. The concentration of each compound tested was 1 mm except for nerol (0.5 mm) and camphor (5 mm). Download Figure 5-1, EPS file.

### Amino acids in the multiple domains of AmHsTRPA are necessary for the response to diallyl disulfide and camphor

**Figure 6.**
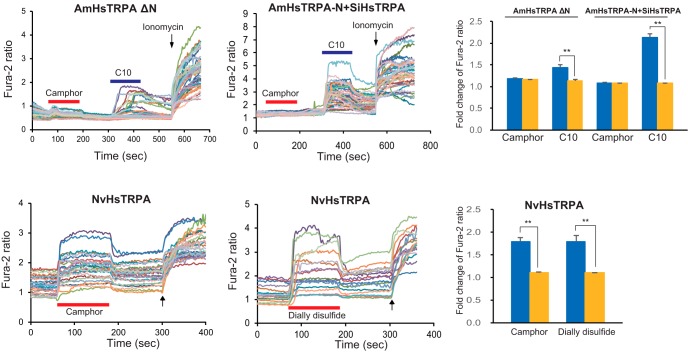
Camphor activates NvHsTRPA but not N-terminal-deleted AmHsTRPA mutant and SiHsTRPA added with N terminus of AmHsTRPA. Responses of N-terminal-deleted AmHsTRPA mutant (AmHsTRPA ΔN), SiHsTRPA added with N terminus of AmHsTRPA (AmHsTRPA-N + SiHsTRPA), and NvHsTRPA to camphor and diallyl disulfide were tested by calcium imaging. Red bars show the period when each compound was added, and then washed off. C10 was applied to the mutants after testing camphor as shown by blue bars. Arrows show the time points when ionomycin was added. The mean value of fold change of Fura-2 ratio with error bar (± SEM) is shown for the compound stimulated DsRed-positive (blue) and -negative (yellow) cells. The total numbers of AmHsTRPA ΔN-, AmHsTRPA-N + SiHsTRPA-, and NvHsTRPA-transfected cells analyzed were 100–114, 104–109, and 46–90, respectively. **, Significant difference by one-tailed Welch’s *t* test (*P* < 0.001). Amino acid sequence alignment of wasp, bee, and ant HsTRPA channels is shown in extended data Fig. 6-1. Molecular phylogenetic tree of wasp, bee, and ant HsTRPA channels is indicated in extended data Fig. 6-2.

Alignment of HsTRPA amino acid sequences from jewel wasp, leaf cutting bee, honey bee, bumble bee, and seven ants revealed that the N-terminal sequences of bee HsTRPA channels are longer than those of the HsTRPA channels of the jewel wasp and ants (extended data Fig. 6-1). It was previously reported that the long N-terminal sequence of the honey bee mite, *Tropilaelaps mercedesae*, TRPA1 (TmTRPA1) isoform plays a critical role in chemical sensitivity. The TmTRPA1 isoform with a short N-terminal sequence responded to fewer compounds (Dong et al., 2016). The same observation was also made with *D. melanogaster* TRPA1 (Kang et al., 2012; Du et al., 2015). We therefore tested the effects of deleting the N-terminal sequence of AmHsTRPA (amino acids 21–98) and adding the sequence (amino acid 1–98) to SiHsTRPA. The N-terminal deletion mutant of AmHsTRPA (AmHsTRPA ΔN) was weakly activated by decanoic acid, but not by 5 mm camphor. Meanwhile, decanoic acid, but not camphor, activated SiHsTRPA with the N-terminal sequence of AmHsTRPA added (AmHsTRPA-N + SiHsTRPA; Fig. 6). These results demonstrate that the N-terminal sequence is necessary but not sufficient to support the full chemical activation of AmHsTRPA. In fact, we found that both camphor and diallyl disulfide robustly activate NvHsTRPA, which lacks the extended N-terminal sequence and outgroups both AmHsTRPA and SiHsTRPA (Fig. 6 and extended data Figs. 6-1 and 6-2). These results suggest that amino acid substitutions in SiHsTRPA have resulted in the loss of reactivity to the above compounds.

10.1523/ENEURO.0327-17.2018.f6-1 Figure 6-1 Amino acid sequence alignment of wasp, bee, and ant HsTRPA channels and the positions of ankyrin repeats, transmembrane segments, and selectivity filter. HsTRPA channels of *N. vitripennis* (jewel wasp), *Megachile rotundata* (leafcutter bee), *Apis mellifera* (western honey bee), *Bombus terrestris* (buff-tailed bumble bee), *Harpegnathos saltator* (Jerdon’s jumping ant), *Camponotus floridanus* (Florida carpenter ant), *Linepithema humile* (Argentine ant), *Pogonomyrmex barbatus* (red harvester ant), *Solenopsis invicta* (red imported fire ant), *Acromyrmex echinatior* (Panamanian leafcutter ant), and *Atta cephalotes* (leafcutter ant) were aligned by MUSCLE. The extended N-terminal sequences of MrHsTRPA, AmHsTRPA, and BtHsTRPA channels are highlighted with gray. Odd and even numbered ankyrin repeats (ARs1–7) in AmHsTRPA and SiHsTRPA are highlighted by yellow and green, respectively. The transmembrane segments (S1–6) and selectivity filter (SF) are highlighted by blue and purple, respectively. Identical amino acids between the 11 channels are indicated by asterisks, and the similar amino acids are shown by either . or :. Download Figure 6-1, DOCX file.

10.1523/ENEURO.0327-17.2018.f6-2 Figure 6-2 Molecular phylogenetic tree of wasp, bee, and ant HsTRPA channels. The evolutionary history of wasp, bee, and ant HsTRPA channels was inferred by using the maximum likelihood method based on the JTT matrix-based model. A discrete gamma distribution was used to model evolutionary rate differences among sites. The tree is drawn to scale, with branch lengths measured in the number of substitutions per site. *Acyrthosiphon pisum* (pea aphid) HsTRPA-like channel (ApHsTRPA-like) was used as the outgroup. Bootstrap values with 500 replications are also shown at each node of the tree. Ant, bee, and wasp HsTRPA channels are indicated by red, blue, and green brackets, respectively. Download Figure 6-2, TIF file.

**Figure 7.**
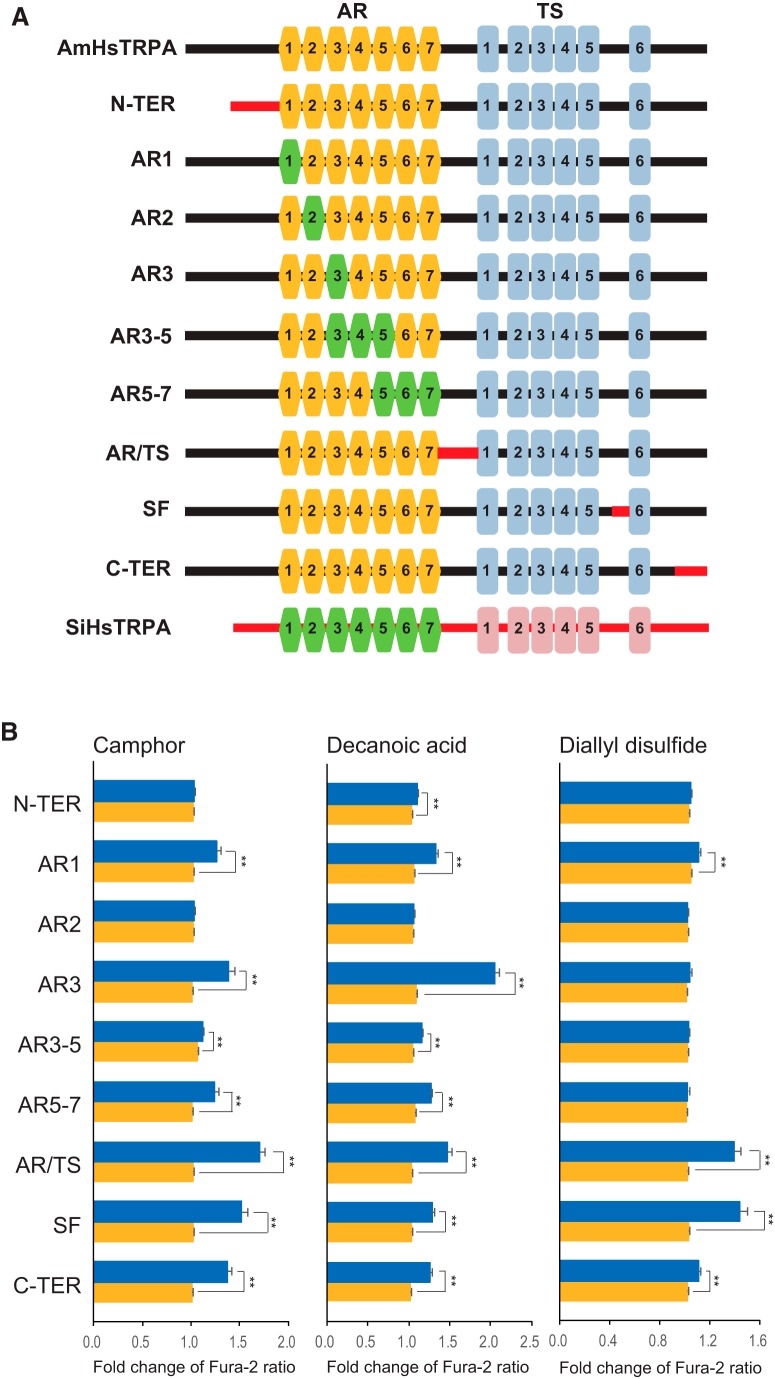
Responses of nine AmHsTRPA/SiHsTRPA chimeric channels to camphor, diallyl disulfide, and decanoic acid. ***A***, Schematic representation of nine AmHsTRPA/SiHsTRPA chimeras as well as the parental AmHsTRPA and SiHsTRPA channels. Ankyrin repeat (AR) and transmembrane segment (TS) are shown by hexagon and rectangle, respectively. All chimeras were based on AmHsTRPA in which the N terminus (N-TER), AR1, AR2, AR3, AR3-5, AR5-7, the boundary between ARs and TS (AR/TS), the selectivity filter (SF), and the C terminus (C-TER) was swapped by the corresponding domain of SiHsTRPA. ***B***, The mean value of fold change of Fura-2 ratio with error bar (± SEM) is shown for the compound-stimulated DsRed-positive (blue) and DsRed-negative (yellow) cells. The total numbers of camphor-, diallyl disulfide-, and decanoic acid-stimulated cells analyzed were 91–124, 87–122, and 180–246, respectively. **, Significant difference by one-tailed Welch’s *t* test (*P* < 0.001). The traces in extended data Fig. 7-1 and 7-2 indicate the changes of intracellular calcium level in cells expressing above AmHsTRPA/SiHsTRPA chimeric channel by camphor, diallyl disulfide, or C10. Protein expression of nine AmHsTRPA/SiHsTRPA chimeric channels is shown in extended data Fig. 7-3.

To identify the protein domains of AmHsTRPA critical for the responses to diallyl disulfide and camphor, we constructed nine AmHsTRPA chimeric channels in which the specific domains with relatively low sequence similarity were swapped between AmHsTRPA and SiHsTRPA (Fig. 7*A*). All chimeric proteins were equally expressed in HEK293 cells (extended data Fig. 7-3), and their responses to the above compounds, as well as decanoic acid, were tested (Fig. 7*B* and extended data Figs. 7-1 and 7-2). Because the reactivity of selectivity filter and C terminus–swapped chimeras to decanoic acid was obscure after stimulation by either diallyl disulfide or camphor (extended data Fig. 7-2), we tested the responses of these two chimeras to decanoic acid alone (extended data Fig. 7-2, bottom panels). Amino acids in the AR2 of AmHsTRPA were necessary for the responses to camphor, diallyl disulfide, and decanoic acid, and the responses of chimeric channels to camphor and decanoic acid were similar (Fig. 7B and extended data Figs. 7-1 and 7-2). Thus, the same protein domains of AmHsTRPA have important roles for responding to the above two compounds. In contrast, amino acids in ARs 3 and 5–7 of AmHsTRPA were specifically required for the response to diallyl disulfide (Fig. 7B and extended data Figs. 7-1 and 7-2). These results suggest that camphor and decanoic acid share the same activation mechanism for AmHsTRPA, which is expectedly different from the mechanism of an electrophile, diallyl disulfide.

10.1523/ENEURO.0327-17.2018.f7-1 Figure 7-1 Responses of nine AmHsTRPA/SiHsTRPA chimeric channels to diallyl disulfide, decanoic acid, and camphor. Responses of nine AmHsTRPA/SiHsTRPA chimeric channels to camphor, diallyl disulfide, and decanoic acid were tested by calcium imaging. Red, green, and blue bars show the period when diallyl disulfide, camphor, and C10 were added, and then washed off, respectively. Arrows show the time points when ionomycin was added. Download Figure 7-1, EPS file.

10.1523/ENEURO.0327-17.2018.f7-2 Figure 7-2 Responses of nine AmHsTRPA/SiHsTRPA chimeric channels to diallyl disulfide, decanoic acid, and camphor. Responses of nine AmHsTRPA/SiHsTRPA chimeric channels to camphor, diallyl disulfide, and decanoic acid were tested by calcium imaging. Red, green, and blue bars show the period when diallyl disulfide, camphor, and C10 were added, and then washed off, respectively. Arrows show the time points when ionomycin was added. Download Figure 7-2, EPS file.

10.1523/ENEURO.0327-17.2018.f7-3 Figure 7-3 Expression of nine AmHsTRPA/SiHsTRPA chimeric channel proteins. The channel proteins (***A***) and β-tubulin (***B***) expressed in HEK293 cells transfected with empty vector (Mock), AmHsTRPA- and AmHsTRPA/SiHsTRPA chimeric channel (N-TER, AR1, AR2, AR3, AR3-5, AR5-7, AR/TS, SF, and C-TER)-expressing constructs were analyzed by Western blot. The size (in kilodaltons) of protein molecular weight marker (MW) is at the left. Download Figure 7-3, TIF file.

### SiHsTRPA-activating compounds repel *S. invicta*

**Figure 8.**
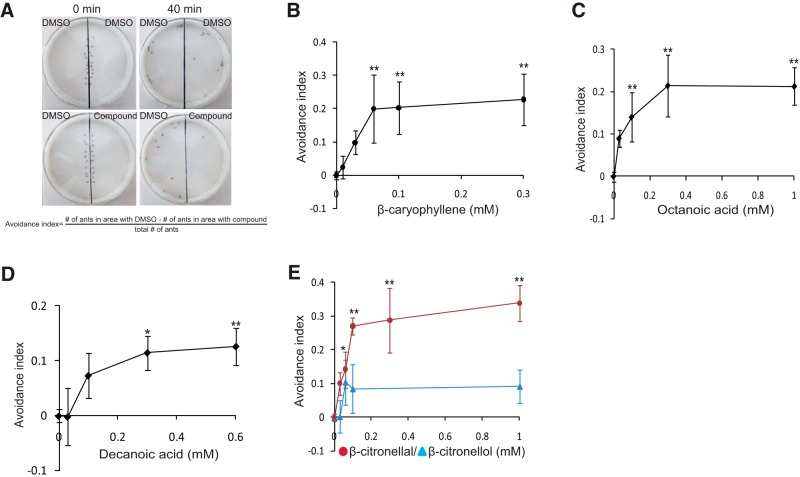
SiHsTRPA-activating compounds repel fire ants. ***A***, We introduced 20 fire ants at the boundary of two filter papers soaked with either compound or 10% DMSO, and then the ants were allowed to move for 40 min. The number of ants in each area was counted every 5 min for 10–40 min, and the seven recordings were summed. In the control experiments, both filter papers were soaked with 10% DMSO. We calculated the avoidance index as shown. The avoidance index is shown at the different concentrations of β-caryophyllene (***B***), octanoic acid (***C***), decanoic acid (***D***), β-citronellal (***E***; purple circle), and β-citronellol (***E***; blue triangle). The mean value with error bar (± SEM; *n* = 3) is indicated for each concentration. **, *P*-values at 0.06, 0.1, and 0.3 mm of β-caryophyllene are <0.0033, <0.0028, and <0.0007, respectively. *P*-values at 0.1, 0.3, and 1 mm of octanoic acid <0.009, <0.0001, and <0.0001. *P*-values at *, 0.3, and **, 0.6 mm, of decanoic acid are <0.02 and <0.004. *P*-values at *, 0.06, and **, 0.1, 0.3, and 1 mm of β-citronellal are <0.02, <7.3 × 10^–6^, < 3.3 × 10^–6^, and <1.4 × 10^–6^. All of above *P*-values are the results of two-tailed Dunnett test compared to 0 mm.

As shown in Fig. 3, SiHsTRPA was activated by cinnamaldehyde and insect repellents, suggesting that it functions as a sensor for noxious compounds. We thus tested whether SiHsTRPA-activating compounds act as repellents for *S. invicta* in the laboratory. We tested the behavior of *S. invicta* placed on two adjacent filter papers soaked with either 10% DMSO or a testing compound in 10% DMSO. In the control assay, both filter papers were soaked with 10% DMSO (Fig. 8*A*). SiHsTRPA-activating compounds, β-caryophyllene (Fig. 8*B*), octanoic acid (Fig. 8*C*), decanoic acid (Fig. 8*D*), and β-citronellal (Fig. 8*E*, purple) repelled *S. invicta* in a concentration-dependent manner. β-Citronellol, which did not activate SiHsTRPA despite its almost identical structure to β-citronellal (Fig. 5), was unable to repel *S. invicta* (Fig. 8*E*, blue). Thus, the repellent activity of a compound reflects its ability to activate SiHsTRPA. These results demonstrate that SiHsTRPA-activating compounds function as repellents against *S. invicta*, and SiHsTRPA is a sensor for noxious compounds.

### Activation of SiHsTRPA is sufficient to modify feeding behavior of *D. melanogaster*

**Figure 9.**
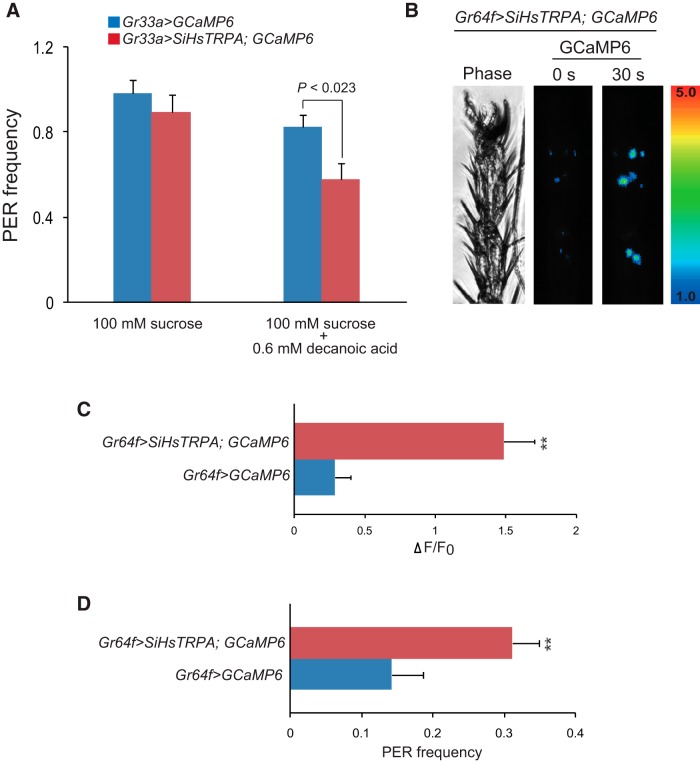
Decanoic acid modifies gustatory responses of *D. melanogaster* expressing SiHsTRPA. ***A***, PER frequency of fruit flies expressing either GCaMP6 (*Gr33a > GCaMP6*) alone or both GCaMP6 and SiHsTRPA (*Gr33a > SiHsTRPA; GCaMP6*) under *Gr33a-Gal4* toward 100 mm sucrose solution containing 0 (*n* = 19–20 for each genotype) or 0.6 mm decanoic acid (*n* = 19–21 for each genotype). The mean value with error bar (± SEM) is shown. PER frequency of fruit flies expressing both GCaMP6 and SiHsTRPA is significantly lower than that of those expressing GCaMP6 alone toward 100 mm sucrose containing 0.6 mm decanoic acid (two-tailed *t* test). ***B***, Phase and GCaMP6 images of fruit fly expressing both SiHsTRPA and GCaMP6 under *Gr64f-Gal4* (*Gr64f > SiHsTRPA; GCaMP6*) before (0 s) and 30 s after applying 0.6 mm decanoic acid to the distal segments of the foreleg. The increase of GCaMP6 fluorescence (ΔF) is indicated by pseudo-color. ***A***, Intracellular calcium changes (Δ*F*/*F*_0_) of SiHsTRPA and GCaMP6- or GCaMP6-expressing sugar taste neuron associated with 5D1 sensilla 30 s after applying 0.6 mm decanoic acid (*n* = 6–9). **, Intracellular calcium level is significantly higher, with fruit flies expressing both SiHsTRPA and GCaMP6 than those expressing GCaMP6 alone (two-tailed *t* test, *P* < 0.0009). ***D***, PER frequency of fruit flies of *Gr64f > SiHsTRPA; GCaMP6* and *Gr64f > GCaMP6* stimulated by 0.6 mm decanoic acid (*n* = 29). **, Fruit flies expressing both GCaMP6 and SiHsTRPA show significantly higher PER frequency than those expressing GCaMP6 alone (two-tailed *t* test, *P* < 0.0049).

To test whether the activation of SiHsTRPA by decanoic acid is sufficient to repress feeding behavior, we characterized the gustatory responses of *D. melanogaster* expressing SiHsTRPA and a calcium-sensor protein, GCaMP6 (Chen et al., 2013) under *Gr33a-Gal4*, to a 100-mm sucrose solution containing 0.6 mm decanoic acid by PER (Kohno et al., 2010; Peng et al., 2015b). *D. melanogaster Gr33a* is a gustatory receptor gene widely expressed in aversive taste neurons (Moon et al., 2009). PER frequency was comparable between fruit flies expressing GCaMP6 alone and both SiHsTRPA and GCaMP6 with 100 mm sucrose. However, it was significantly reduced in the presence of 0.6 mm decanoic acid for the animals expressing both SiHsTRPA and GCaMP6 (Fig. 9*A*). These results demonstrate that in *D. melanogaster*, direct chemical activation of SiHsTRPA in aversive taste neurons could suppress feeding behavior elicited by sucrose.

We also tested whether stimulation of SiHsTRPA in the sugar taste neurons by 0.6 mm decanoic acid was sufficient to induce feeding behavior. We expressed both SiHsTRPA and GCaMP6 in Gr64f-positive sugar taste neurons (Jiao et al., 2008), and GCaMP6 fluorescence was detected in several discrete neurons in the distal segments of the foreleg (Peng et al., 2015b). As shown in Fig. 9*B*, applying 0.6 mm decanoic acid to the distal segments of foreleg increased GCaMP6 fluorescence in the Gr64f-expressing sugar taste neurons associated with multiple sensilla, including 5D1, 5V1, and 5V2 sensilla (Miyamoto et al., 2013). We detected an increase in intracellular calcium level (Δ*F*/*F*_0_) in the sugar taste neuron associated with 5D1 sensilla expressing both SiHsTRPA and GCaMP6, but not expressing GCaMP6 alone (Fig. 9*C*). SiHsTRPA stimulation by decanoic acid was able to activate the sugar taste neurons, and as a result, the application of 0.6 mm decanoic acid to the forelegs was sufficient to induce significant PER in the fruit flies expressing both SiHsTRPA and GCaMP6, but not GCaMP6 alone (Fig. 9*D*). These results demonstrate that manipulating the activity of taste neurons by the introduction of SiHsTRPA and by the application of decanoic acid is sufficient to modify the gustatory behaviors of fruit flies.

## Discussion

### *S. invicta* highly expresses SiHsTRPA in the antennae and legs

We first identified a *Wtrw* ortholog and its duplicated gene, *SiHsTRPA*, in an *S. invicta* genomic scaffold. Consistent with the lack of introns in the *SiHsTRPA* gene, we isolated the single cDNA that encodes a TRPA channel with seven ARs and an ion-transport domain containing six transmembrane segments. These structural features appear to be shared between HsTRPA channels of bees, wasps, and ants. Nevertheless, duplicated *Wtrw* genes are also present in *Bombyx mori* (silk moth), *Manduca sexta* (tobacco hornworm), and *Acyrthosiphon pisum* (pea aphid) genomes, suggesting that the duplication occurred in the common ancestor of insects but the duplicated gene has been lost in many insect species. The retention of HsTRPA in Hymenoptera would be essential because it has to replace the functions of the lost ancient TRPA1 (Matsuura et al., 2009; Kohno et al., 2010). *SiHsTRPA* mRNA is likely to be expressed in sensory neurons associated with various types of sensilla (Renthal et al., 2003; Ramirez-Esquivel et al., 2014). These results may suggest that SiHsTRPA functions as a peripheral sensor to detect stimuli in the environment based on our *in vitro* and heterologous expression experiments. Thus, SiHsTRPA appears to play a major role for sensory perception through sensilla in the antennae and legs.

### SiHsTRPA functions as a heat sensor

Both SiHsTRPA and DmTRPA1 are heat sensitive and share similar heat-associated physiological functions. Nevertheless, heat-sensitive SiHsTRPA did not completely rescue the impaired thermal nociception behavior of *trpA1* mutant larvae. This may suggest that DmTRPA1 plays other roles in addition to a thermal sensor for the thermal nociception behavior as previously reported (Zhong et al., 2012). Considering complex behaviors of ants, they should require thermosensors to avoid extreme temperatures, choose the preferred temperatures, and monitor the nest temperature for brood care. Some species may use temperature information as the cue for other behaviors (Ruchty et al., 2010b). Sensilla ampullaceal, sensilla coeloconica, and coelocapitular sensilla are considered to contain thermosensitive neurons (Ramirez-Esquivel et al., 2014); however, only cold-sensitive neurons have been identified in any ant to date (Ruchty et al., 2010a; Nagel and Kleineidam, 2015). Because several warm-sensitive glomeruli were identified in the antennal lobe of the leaf-cutting ant (Ruchty et al., 2010b), heat-sensitive neurons expressing SiHsTRPA are likely to be present in *S. invicta*.

### Evolutionary plasticity of the HsTRPA channel

Interestingly, NvHsTRPA is activated by camphor and diallyl disulfide, which are incapable of activating SiHsTRPA. These results suggest that AmHsTRPA and NvHsTRPA share a common chemical sensitivity, and based on the molecular species tree of wasps, bees, and ants (Gadau et al., 2012), as well as the phylogenetic tree of HsTRPA channels, SiHsTRPA appears to have lost most of the chemical sensitivity of the common ancestor of HsTRPA. In fact, a number of repellents for *S. invicta* and other ants have been identified previously (Dani et al., 1996; Wheeler et al., 2003; Dutcher and Beaver, 2005; Chen et al., 2008; Kafle and Shih, 2013; Zhang et al., 2014; Fu et al., 2015; Huang et al., 2015), but we found that most of them did not activate SiHsTRPA (Table 1). Several examples are camphor, eugenol, cineole, nerolidol, butyl carbitol acetate, linalool, citral, and limonene. The loss of chemical reactivity of SiHsTRPA might be compensated by the increased number of Pain and/or TRPA5 genes in the *S. invicta* genome (Peng et al., 2015a) or other sensors/receptors. Thus, this is another example that shows the evolutionary plasticity of TRP channels (Kadowaki, 2015; Saito and Tominaga, 2015).

The responses of AmHsTRPA/SiHsTRPA chimeric channels to diallyl disulfide, camphor, and decanoic acid provide insights into the mechanisms for activation of parent channels by these compounds. Although decanoic acid activated both AmHsTRPA and SiHsTRPA, it did not appear to fully activate the N terminus, AR2, and AR4 swapped chimeras. These results suggest that the amino acids in above protein domains have coevolved and thus closely interact with each other in the three-dimensional structure (Ovchinnikov et al., 2015). The protein structure formed by the above three domains, and perhaps other domains (for example, the transmembrane segments) would be responsible for interacting with decanoic acid and gating the channel. Alternatively, it would be involved in opening the gate after decanoic acid interacts with the other domain. Intriguingly, the same protein structure is also responsible for activation by camphor; however, this effect is specific to AmHsTRPA. Considering fewer domains are involved in the activation by decanoic acid/camphor than by diallyl disulfide, we favor a hypothesis that the above protein structure may interact with both decanoic acid and camphor in AmHsTRPA, but only with decanoic acid in SiHsTRPA, and allow opening of the channel gate.

Both cinnamaldehyde and diallyl disulfide were shown to act as electrophiles to covalently modify cysteines in the N terminus of TRPA1 (Hinman et al., 2006; Macpherson et al., 2007); however, SiHsTRPA was activated only by cinnamaldehyde, suggesting that the activation mechanisms of TRPA subfamily members by electrophiles are different. Comparing amino acid sequences of AmHsTRPA and SiHsTRPA, 11 cysteines are conserved, but they also contain eight and six species-specific cysteines, respectively. Cinnamaldehyde may target the conserved cysteines, and diallyl disulfide would modify only AmHsTRPA-specific cysteine residues. However, the mechanism to activate AmHsTRPA by electrophiles cannot be simply explained by the cysteine modifications. For example, amino acids in ARs 3 and 5–7 of AmHsTRPA are essential for activation by diallyl disulfide; nevertheless, cysteine is absent in ARs 3 and 6, and ARs 5 and 7 contain only conserved cysteines. These results suggest that these ARs have important roles for channel activation, other than some of the consisting cysteines being covalently modified. SiHsTRPA has undergone amino acid substitutions in multiple domains and lost reactivity to camphor and diallyl disulfide as a result. Such amino acid substitutions changing the channel’s sensitivity to chemicals may be the result of natural selection, possibly linked to the different ecological niches of these ants relative to those of bees and wasps. It is surprising that SiHsTRPA has undergone these evolutionary changes as yet by maintaining its heat sensitivity. Intriguingly, both β-citronellal (with an aldehyde group) and β-citronellol (with a hydroxyl group) can activate AmHsTRPA, but SiHsTRPA is only activated by β-citronellal. This may suggest that SiHsTRPA activation requires not only binding with β-citronellal but also the modification of an amino group by the aldehyde group.

### Activation of TRPA channels by fatty acids

We found that saturated fatty acids with more than eight carbons (medium-chain fatty acids) activate both SiHsTRPA and AmHsTRPA. Because short-chain fatty acids (C ≤7) failed to activate SiHsTRPA, activation is likely to require plasma membrane penetration of a fatty acid (Kamp and Hamilton, 2006), but not extracellular protons being released from the fatty acid. Activation of human TRPA1 by octanoic acid, nonanoic acid, decanoic acid, and lauric acid was previously reported (Terada et al., 2011); however, another report showed that mouse and rat TRPA1 channels are activated only by fatty acids with >18 carbons and at least three double bonds, and activation by polyunsaturated fatty acids is specific to mammalian TRPA1 channels (Motter and Ahern, 2012). The above discrepancy could be because of species-specific activation (primate vs. rodent TRPA1 channels). How fatty acids activate the above TRPA channels remains to be answered. A local increase in protons released from the incorporated fatty acid may activate the channel. Furthermore, the carbonyl group generated after the release of a proton may act as an electrophilic agent to covalently modify the channel. Alternatively, interaction of a fatty acid with the transmembrane segments together with the other domains may result in activation of the TRPA channel. Motter and Ahern (2012) suggested that mouse TRPA1 is unlikely to be activated by modification of the integrity and/or fluidity of the plasma membrane by a polyunsaturated fatty acid. However, another study suggested that lipopolysaccharide activates mouse TRPA1 through mechanical alterations in the plasma membrane, since the effects are highly dependent on the structure of lipid A (Meseguer et al., 2014).

### Potential use of SiHsTRPA-activating compounds to control *S. invicta*

Although a number of chemical and biological methods have been developed to control *S. invicta* in invaded areas, none of them have been very effective (for example, Collins and Callcott, 1995). Because the SiHsTRPA-activating compounds we identified are natural products that repel *S. invicta*, they could be used to develop such methods. These repellents would also be useful for quarantine treatment, for example, to clear *S. invicta* from imported products at a port. However, the specificity of an effect is a major issue to be addressed. All of the SiHsTRPA-activating compounds activate AmHsTRPA, and perhaps other ant HsTRPA channels, as well as arthropod TRPA1 channels (Du et al., 2015; Peng et al., 2015b; Dong et al., 2016). Screening more compounds may identify those that specifically act on SiHsTRPA. The appropriate use of such natural compounds derived from plants may serve as an alternative method to control *S. invicta.*

## Synthesis

Reviewing Editor: Anna Menini, SISSA (International School of Advances Studies) Decisions are customarily a result of the Reviewing Editor and the peer reviewers coming together and discussing their recommendations until a consensus is reached. When revisions are invited, a fact-based synthesis statement explaining their decision and outlining what is needed to prepare a revision will be listed below. The following reviewer(s) agreed to reveal their identity: Paul Heppenstall, Jean-Christophe Sandoz.

The manuscript characterizes the first Hymenoptera-specific TRPA channel of an ant species, the red imported fire ant. The authors use a multitude of approaches to characterize the channel and demonstrate specializations in its sensitivity to plant-derived chemicals, to pinpoint the responsible protein domains, and to show that it may be a viable molecular target for pest control.

Both reviewers agree that the experimental approach is elegant and that the experiments appear to be well performed. However, because of the broad number of techniques employed in the study and the large amount of data, the manuscript is somehow difficult to follow and will benefit from better labeling of the figures, additional details and some reorganization. The authors should make appropriate revisions to the manuscript according to the following comments:

Major points:

1) Statistics are provided for all experiments except for the HEK cell imaging experiments, in which decision if there is a response or not is totally opaque and qualitative. The authors must calculate response amplitudes and provide statistics allowing to make a decision whether or not a given drug induces a response. Graphs cannot only present a bunch of individual traces and give no indication of the number of cells measured and, most importantly, the number of times this particular experiment was run. Do all traces belong to the same dish and to only one drug delivery? Please add mean values and error bars for all these experiments. Presenting this data correctly is necessary for readers to actually believe in the data.

2) Thermotactic behavior experiment: the description of this experiment is really unclear.

What does “the positions in the apparatus were stabilized” mean (L245)? From the text, it is difficult to understand what was exactly the criteria for stopping the experiment. The authors write that there was no difference in the time spent by the different groups in the system, but real data/analysis showing that are missing. Lastly, the authors mention that they made photos every 10 min, but it is not explained how this is used in the data presented in Fig 2B. All this should be better explained. It is also crucial to state clearly that fruit flies' behaviour is the product of both SiHsTRPA expressed by the authors AND their own thermosensitive channels.

Other points:

-The Abstract is overly complicated and should be simplified.

-The Significance Statement only recapitulates the Abstract and does not contain much significance. Please revise it. Indeed, the Introduction contains more text on the significance of the study. In addition, while the language is mostly good in the main manuscript (but see corrections below), that of the Significance Statement is suboptimal. It should be revised by a native speaker.

-Figure 2B: there is a typo in temperature.

-Figure 2C: use same scale for all graphs (the blue one should go to 80% too).

-Figure 3 and ff: sometimes the chemical structure of the drug is given, but most of the time it is not. Why is it so? Please unify.

-Figures 3-8: why are not all cell imaging assays presented with the same scale, so that readers can interpret differences in response amplitude correctly. Also mean values and error bars are missing in all these experiments.

-Legends of Figures 3 to 8: indicate number of replicates and numbers of cells for each experiment.

-Figure 6: it would be better to show the SiHsTRPA1 data first as this is the focus of the study.

-Figure 7: a better example for AmHsTRPA-N-SiHsTRPA1 should be shown as it seems there is some spontaneous activity prior to adding Camphor.

-Figure 8: drug names are often missing where they would be useful.

-Figure 9: X min should be defined on the figure.

-Figure 10A: legend of Y axis is missing

-PER should be redefined again in the results section.

-For the expression analysis of mutation channels in the supplementary information, a loading control should be included.

-The discussion could be toned down a little, for example by replacing the word “must” with “may”.

L40: space missing after “environment”

L43: “more rapid evolution than several...”

L73: “and pyrethroids”

L223: “to deliver hot bath solution”. I would add “(for temperature stimulations)”

L226: please describe the imaging system used, as well as how the imaging data were analyzed

L243: “middle of the testing arena”

L260: “The PER assay”

L270: description of the PER assay is difficult to follow, especially because at this stage, the reader has no idea why the authors use decanoic acid. Please explain why decanoic acid should be accepted by flies here, etc.

L276: avoid “as described previously”. Please think about the readers of this paper and describe the experiment correctly.

L282: “Repellent... in the laboratory”

L285: “beginning of the assay”

L287: “in the area”. Please define this area. What shape did it have?

L352: this conclusion is unclear. If I understand well, the authors want to explain that SiHsTRPA was expressed in fruitflies in addition to their own thermosensitive channels and that the behaviour recorded in this assay is a function of all these channels. This is indeed an important point to make, but this should be rephrased.

L354: change title to: “...show different chemical responses”

L355 and ff: see major comment on HEK cell experiments. The reader needs to know how many cells were imaged and how many replicates were done. They also need clear statistics about each response.

L369-370: parenthesis. Why not list the compounds in the chain length order? or indicate why this order was chosen

L379: “with a hydroxyl”

L419: “act as repellents”

L424: “Intriguingly” is misleading here. What is intriguing is the fact that b-citronellal but not b-citronellol activated SiHsTRPA, not what is said in the sentence. If b-citronellol does not activate the channel then it is only logical that it does not repel the ants.

L437: more explanations are needed here. I don't see why if C22:6 does not activate DmTRPA1, decanoic acid should not.

L439: “it was significantly reduced in the presence of... for the animals expressing...”

L443-L456: I'm really wondering what this experiment really brings to the paper. It looks more like a “scientific circus display” than a real experiment. Is HsTRPA ever expressed in gustatory neurons? In the absence of real scientific arguments, the experiment looks only like playing around with transgenic animals.

L442-444: The terms “gustatory avoidance” and “gustatory attraction” are misleading, since the animals do not move.

L472 and elsewhere: “play a major role”

L471: this conclusion is a bit strong. It should be stated in the discussion that this conclusion is based only on in vitro and heterologous expression experiments. The authors never actually blocked activity of SiHsTRPA in ants.

L475-476: This part is unclear. Why do the authors say that? They should explain why experiments in the present MS allow them to say that “DmTRPA1 does not simply function as a thermal sensor for nociception”. As it is, most readers won't understand the point made.

L490: “most of the chemical sensitivity”

L502 and 507: “interact with”

L512: “the channel gate”?

L520: “cannot”

L521: “ARs 3”

L530: “ants inhabit a specific ecological niche” is a bit oversimplifying. There are 20,000 species of ants.

L561: “useful for quarantine treatment”. Please explain how.

Table 1: it would be nice to also have the relative efficacy of the different components for opening the channels in this table. A relative calcium response amplitude maybe?

L776: “using a 46{degree sign}C probe”

## Author Response

Synthesis of Reviews:

Synthesis Statement for Author (Required):

The manuscript characterizes the first Hymenoptera-specific TRPA channel of an ant species, the red imported fire ant. The authors use a multitude of approaches to characterize the channel and demonstrate specializations in its sensitivity to plant-derived chemicals, to pinpoint the responsible protein domains, and to show that it may be a viable molecular target for pest control.

Both reviewers agree that the experimental approach is elegant and that the experiments appear to be well performed. However, because of the broad number of techniques employed in the study and the large amount of data, the manuscript is somehow difficult to follow and will benefit from better labeling of the figures, additional details and some reorganization. The authors should make appropriate revisions to the manuscript according to the following comments:

Major points:

1) Statistics are provided for all experiments except for the HEK cell imaging experiments, in which decision if there is a response or not is totally opaque and qualitative. The authors must calculate response amplitudes and provide statistics allowing to make a decision whether or not a given drug induces a response. Graphs cannot only present a bunch of individual traces and give no indication of the number of cells measured and, most importantly, the number of times this particular experiment was run. Do all traces belong to the same dish and to only one drug delivery? Please add mean values and error bars for all these experiments. Presenting this data correctly is necessary for readers to actually believe in the data.

Authors: Each calcium-imaging experiment was repeated twice except three times for stimulation of SiHsTRPA by heat or β-citronellol and stimulation of AmHsTRPA by octanoic acid. We calculated the fold change of Fura-2 ratio (the maximum Fura-2 ratio recorded after the stimulation divided by Fura-2 ratio just prior to the stimulation) in the individual DsRed-positive (with the channel expression) and -negative (without the channel expression) cells. This corresponds to the change of intracellular calcium concentration in the cell with or without the channel by the specific stimulation and we statistically compared the values between DsRed-positive and -negative cells. This has been described in Line 224-229 of the revised manuscript. The mean values of fold changes of Fura-2 ratio with error bars ({plus minus} SEM) were shown for the temperature/compound stimulated DsRed-positive and -negative cells. The total numbers of cells analyzed were also indicated in the figure legends.

2) Thermotactic behavior experiment: the description of this experiment is really unclear.

What does “the positions in the apparatus were stabilized” mean (L245)? From the text, it is difficult to understand what was exactly the criteria for stopping the experiment. The authors write that there was no difference in the time spent by the different groups in the system, but real data/analysis showing that are missing. Lastly, the authors mention that they made photos every 10 min, but it is not explained how this is used in the data presented in Fig 2B. All this should be better explained. It is also crucial to state clearly that fruit flies' behaviour is the product of both SiHsTRPA expressed by the authors AND their own thermosensitive channels.

Authors: Fruit flies moved very rapidly in the entire area when they were first introduced into the apparatus; however, they gradually ceased rapid movement in the apparatus. We then counted the number of fruit flies in each observation field and divided by the total number of fruit flies in the entire area every 10 min between 1.5 and 2.5 h after the introduction. The average value of six recordings was calculated for each experimental group. This has been explained in Line 246-251 of the revised manuscript. The recording was repeated three times for each group as previously described in the legend of Figure 2. We agree that thermotactic behavior of adult D. melanogaster would depend on not only DmTRPA1 but also other thermo-sensors such as Pyx and Gr28b (Lee et al., 2005; Ni et al., 2013). This has been described in Line 345-347 of the revised manuscript.

Other points:

-The Abstract is overly complicated and should be simplified.

Authors: We have simplified the Abstract as much as possible in the revised manuscript as suggested (Line 2-17).

-The Significance Statement only recapitulates the Abstract and does not contain much significance. Please revise it. Indeed, the Introduction contains more text on the significance of the study. In addition, while the language is mostly good in the main manuscript (but see corrections below), that of the Significance Statement is suboptimal. It should be revised by a native speaker.

Authors: In the revised manuscript, we have added the Significance Statement which was edited by a native speaker, the Head of Department of Biological Sciences, Xi'an Jiaotong-Liverpool University (Line 21-29).

-Figure 2B: there is a typo in temperature.

Authors: It has been corrected in Figure 2B of the revised manuscript.

-Figure 2C: use same scale for all graphs (the blue one should go to 80% too).

Authors: We have shown the graph (ppk-1 < SiHsTRPA; trpA11) with the same scale as others in a new Figure 2 of the revised manuscript.

-Figure 3 and ff: sometimes the chemical structure of the drug is given, but most of the time it is not. Why is it so? Please unify.

Authors: Structures of β-citronellal and β-citronellol have been removed from the figures in the revised manuscript.

-Figures 3-8: why are not all cell imaging assays presented with the same scale, so that readers can interpret differences in response amplitude correctly. Also mean values and error bars are missing in all these experiments.

Authors: As shown in the traces of all calcium-imaging data, the intracellular calcium level varied extensively in the individual cells after adding ionomycin. Furthermore, the maximum Fura-2 ratio obtained was quite different between the experiments. We think this happened because we roughly set Fura-2 ratio to 1 at the beginning of each assay by adjusting exposure time for 340 nm and 380 nm excitation. This adjustment most likely depends on the initial intracellular calcium levels in the cells which are difficult to control for each experiment. Thus, to make the changes of Fura-2 ratio clearly visible, the graph containing Y-axis with the different scale was shown for each experiment. However, as explained above for the Major point 1), we have shown the mean values of fold changes of Fura-2 ratio with error bars ({plus minus} SEM) for the temperature/compound stimulated DsRed-positive and -negative cells in the revised manuscript. We have focused on the differences between DsRed-positive and -negative cells which were assayed under the same condition. It is difficult to compare the results of different experiments unless they were repeated many times. The mean value of fold changes of Fura-2 ratio in the cells expressing SiHsTRPA or AmHsTRPA induced by each compound is also shown in Table 1 of the revised manuscript.

-Legends of Figures 3 to 8: indicate number of replicates and numbers of cells for each experiment.

Authors: Please refer to our response to the Major point 1) above.

-Figure 6: it would be better to show the SiHsTRPA1 data first as this is the focus of the study.

Authors: We have changed the order in Extended data Figure 5-1 of the revised manuscript.

-Figure 7: a better example for AmHsTRPA-N-SiHsTRPA1 should be shown as it seems there is some spontaneous activity prior to adding Camphor.

Authors: We have replaced it with the new trace in Figure 6 of the revised manuscript.

-Figure 8: drug names are often missing where they would be useful.

Authors: Names of compounds have been added to Extended Figures 7-1 and 7-2 of the revised manuscript.

-Figure 9: X min should be defined on the figure.

Authors: The image was photographed at 40 min after the start of experiment so that X has been changed to 40 in Figure 8 of the revised manuscript.

-Figure 10A: legend of Y axis is missing

Authors: We have added “PER frequency” as legend of Y-axis in Figure 9A of the revised manuscript.

-PER should be redefined again in the results section.

Authors: We have redefined PER in the Results section of the revised manuscript as suggested (Line 446).

-For the expression analysis of mutation channels in the supplementary information, a loading control should be included.

Authors: We analyzed the cell lysates containing the chimeric channels by western blot using anti-β-tubulin antibody to confirm the equal amount of protein was loaded in each sample. This has been shown as a panel B in Extended data Figure 7-3 of the revised manuscript.

-The discussion could be toned down a little, for example by replacing the word “must” with “may”.

Authors: We have revised the Discussion section of the revised manuscript accordingly.

L226: please describe the imaging system used, as well as how the imaging data were analyzed

Authors: The Fura-2 ratio (340/380 nm) were acquired and recorded every 3 second by IPlab software (Scanalytics Inc.). Changes of Fura-2 ratio by time were analyzed by Image-J. This has been described in Line 222-224 of the revised manuscript. We have further analyzed all calcium-imaging data as explained in our response to the Major point 1) above.

L270: description of the PER assay is difficult to follow, especially because at this stage, the reader has no idea why the authors use decanoic acid. Please explain why decanoic acid should be accepted by flies here, etc.

Authors: We have added “to activate SiHsTRPA” following “0.6 mM decanoic acid (WAKO)” in Line 269-270 of the revised manuscript. We observed the fruit flies always showed normal PER to the first offering of 100 mM sucrose with 0.6 mM decanoic acid. This is perhaps because the first activation of aversive taste neurons by SiHsTRPA was not sufficient to repress proboscis extension induced by activation of sugar taste neurons with 100 mM sucrose. This has been described in Line 275-276 of the revised manuscript.

L276: avoid “as described previously”. Please think about the readers of this paper and describe the experiment correctly.

Authors: We have explained the detail of experiment in Line 283-287 of the revised manuscript.

L287: “in the area”. Please define this area. What shape did it have?

Authors: We have explained this in Line 296 of the revised manuscript.

L352: this conclusion is unclear. If I understand well, the authors want to explain that SiHsTRPA was expressed in fruitflies in addition to their own thermosensitive channels and that the behaviour recorded in this assay is a function of all these channels. This is indeed an important point to make, but this should be rephrased.

Authors: SiHsTRPA was expressed in trpA1 mutant background but other endogenous thermosensitive channels are present as pointed out. Pain and Pyx were previously shown to be involved in the thermal nociception behavior of fruit fly larvae. We thus modified the sentence to “These results suggest that SiHsTRPA could complement the functions of DmTRPA1 together with Pain and Pyx (Tracey et al., 2003; Neely et al., 2011; Zhong et al., 2012) for thermal nociceptive behavior in fruit fly larvae” in the revised manuscript (Line 363-366).

L355 and ff: see major comment on HEK cell experiments. The reader needs to know how many cells were imaged and how many replicates were done. They also need clear statistics about each response.

Authors: Please refer to our response to Major point 1) above.

L369-370: parenthesis. Why not list the compounds in the chain length order? or indicate why this order was chosen

Authors: We have changed the order as suggested in the revised manuscript (Line 382-383).

L424: “Intriguingly” is misleading here. What is intriguing is the fact that b-citronellal but not b-citronellol activated SiHsTRPA, not what is said in the sentence. If b-citronellol does not activate the channel then it is only logical that it does not repel the ants.

Authors: We have removed “Intriguingly” from the sentence in the revised manuscript (Line 437) as suggested.

L437: more explanations are needed here. I don't see why if C22:6 does not activate DmTRPA1, decanoic acid should not.

Authors: If DmTRPA1 can be activated by modification of the integrity and/or fluidity of the plasma membrane, docosahexaenoic acid (C22:6) should be more potent than decanoic acid (C10:0) since it is able to mechanically alter the plasma membrane more drastically. However, we have not directly tested the effects of decanoic acid on all isoforms of DmTRPA1, and thus this sentence has been removed from the revised manuscript (Line 448).

L443-L456: I'm really wondering what this experiment really brings to the paper. It looks more like a “scientific circus display” than a real experiment. Is HsTRPA ever expressed in gustatory neurons? In the absence of real scientific arguments, the experiment looks only like playing around with transgenic animals.

Authors: As pointed out, we have no evidence to indicate SiHsTRPA plays any roles for feeding behavior of fire ant. However, we wanted to show SiHsTRPA and its activators or heat could be used as new tool to manipulate the neuronal activity in a small animal such as fruit fly by these experiments.

L442-444: The terms “gustatory avoidance” and “gustatory attraction” are misleading, since the animals do not move.

Authors: Above terms have been replaced by “to repress feeding behavior” and “to induce feeding behavior”, respectively in the revised manuscript (Line 442, 443, 452, and 455).

L471: this conclusion is a bit strong. It should be stated in the discussion that this conclusion is based only on in vitro and heterologous expression experiments. The authors never actually blocked activity of SiHsTRPA in ants.

Authors: The sentence has been changed to “These results may suggest SiHsTRPA functions as a peripheral sensor to detect stimuli in the environment based on our in vitro and heterologous expression experiments.” in the revised manuscript as suggested (Line 481-483).

L475-476: This part is unclear. Why do the authors say that? They should explain why experiments in the present MS allow them to say that “DmTRPA1 does not simply function as a thermal sensor for nociception”. As it is, most readers won't understand the point made.

Authors: As reported in Zhong et al. 2012 paper, the class IV multidendritic nociceptive neurons of fruit fly larva express the heat-insensitive isoform of DmTRPA1 which is capable of rescuing the impaired thermal nociception behavior of trpA1 mutant larvae. The authors thus suggested DmTRPA1 may not simply function as the thermal sensor but perhaps have other roles in the mdIV neurons. Our result that SiHsTRPA did not fully rescue the impaired thermal nociception behavior of trpA1 mutant larvae would be consistent with above hypothesis. We have modified the sentence to “Nevertheless, heat-sensitive SiHsTRPA did not completely rescue the impaired thermal nociception behavior of trpA1 mutant larvae. This may suggest that DmTRPA1 has other role in addition to a thermal sensor for the thermal nociception behavior as previously reported (Zhong et al., 2012).” in the revised manuscript (Line 487-490).

L530: “ants inhabit a specific ecological niche” is a bit oversimplifying. There are 20,000 species of ants.

Authors: The sentence has been changed to “Such amino acid substitutions may have changed the channel to respond to compounds different from AmHsTRPA/NvHsTRPA activators since an ecological niche of ants differs from the ones of bees and wasps.” in the revised manuscript accordingly (Line 539-541).

L561: “useful for quarantine treatment”. Please explain how.

Authors: New invasion of fire ants often starts from a port where imported products are landed first. SiHsTRPA-activating compounds are able to repel fire ants, and thus these compounds could be used to clear fire ants from the imported products at the port. This has been described in the revised manuscript (Line 571-572).

Table 1: it would be nice to also have the relative efficacy of the different components for opening the channels in this table. A relative calcium response amplitude maybe?

Authors: The mean value of fold changes of Fura-2 ratio in the cells expressing SiHsTRPA or AmHsTRPA induced by each activating compound (reported in this paper) has been shown in Table 1 of the revised manuscript accordingly.

L40: space missing after “environment”

L43: “more rapid evolution than several...”

L73: “and pyrethroids”

L223: “to deliver hot bath solution”. I would add “(for temperature stimulations)”

L243: “middle of the testing arena”

L260: “The PER assay”

L282: “Repellent... in the laboratory”

L285: “beginning of the assay”

L354: change title to: “...show different chemical responses”

L379: “with a hydroxyl”

L419: “act as repellents”

L439: “it was significantly reduced in the presence of... for the animals expressing...”

L472 and elsewhere: “play a major role”

L490: “most of the chemical sensitivity”

L502 and 507: “interact with”

L512: “the channel gate”?

L520: “cannot”

L521: “ARs 3”

L776: “using a 46{degree sign}C probe”

Authors: All of above spelling and grammatical errors pointed out by the reviewer have been corrected in the revised manuscript.
