# Supplementary material for: HsTRPA of the Red Imported Fire Ant, Solenopsis invicta, Functions as a Nocisensor and Uncovers the Evolutionary Plasticity of HsTRPA Channels
Source: eNeuro. 2018 Feb 6;5(1):ENEURO.0327-17.2018. doi: 10.1523/ENEURO.0327-17.2018 (PMC5810042; doi:10.1523/ENEURO.0327-17.2018)
Supplement: Figure 6-1 — Amino acid sequence alignment of wasp, bee, and ant HsTRPA channels and the positions of ankyrin repeats, transmembrane segments, and selectivity filter. HsTRPA channels of N. vitripennis (jewel wasp), Megachile rotundata (leafcutter bee), Apis mellifera (western honey bee), Bombus terrestris (buff-tailed bumble bee), Harpegnathos saltator (Jerdon’s jumping ant), Camponotus floridanus (Florida carpenter ant), Linepithema humile (Argentine ant), Pogonomyrmex barbatus (red harvester ant), Solenopsis invicta (red imported fire ant), Acromyrmex echinatior (Panamanian leafcutter ant), and Atta cephalotes (leafcutter ant) were aligned by MUSCLE. The extended N-terminal sequences of MrHsTRPA, AmHsTRPA, and BtHsTRPA channels are highlighted with gray. Odd and even numbered ankyrin repeats (ARs1–7) in AmHsTRPA and SiHsTRPA are highlighted by yellow and green, respectively. The transmembrane segments (S1–6) and selectivity filter (SF) are highlighted by blue and purple, respectively. Identical amino acids between the 11 channels are indicated by asterisks, and the similar amino acids are shown by either . or :. Download Figure 6-1, DOCX file. [file sup_enu-eN-NWR-0327-17-s03.docx]

NvHsTRPA ------------------------------------------------------------

MrHsTRPA ----------------MNVLGPTIVTEERPRRYSLTSLRDRRRSFD-ENDCWSAIDGDGD

AmHsTRPA MDDKQIHENSTDIPKASLQLPTITVGNERPRRYSLTSLKDRRLPFENENDCGRAVEGEVE

BtHsTRPA MDDKQMYENSSDIPKINVQRATIKVSEERLRRYSLTSLKDCRLSVD-EKDYWRTIEGDAG

HsHsTRPA ------------------------------------------------------------

CfHsTRPA ------------------------------------------------------------

LhHsTRPA ------------------------------------------------------------

PbHsTRPA ------------------------------------------------------------

SiHsTRPA ------------------------------------------------------------

AeHsTRPA ------------------------------------------------------------

AcHsTRPA ------------------------------------------------------------

NvHsTRPA -------------------------------------MSRSWKLDEVVEEDPSDRVDMSD

MrHsTRPA MHKNMDKASQTKIKRLTSRSRRTRHYSLTRLKNRRPF-CFDDDRDS--SPKTDNTVTDQM

AmHsTRPA AHENVDKTWQMKMKPLANHEKRTRHYSLTRSKNRRSCLYTDEDRNFLTKSVVNETVNDKT

BtHsTRPA AYENVEKTSQSKMKPLANHKKRTRHYSLTRLRNRRSCLNFDDDKNILTKTSVNETAKDKT

HsHsTRPA ----------------------------------------MDERN---LRNANAKV-DLE

CfHsTRPA --------------------------------------MHKNERDS--VEE-AKNTNDLK

LhHsTRPA --------------------------------------MCENEREGKLSEEKKAKIEKLE

PbHsTRPA --------------------------------------MYENDRDS--LEEKSIKSKDLE

SiHsTRPA --------------------------------------MCENDKDS--LGD-SAKVK--E

AeHsTRPA --------------------------------------MYENDRDS--LGE-SAKVKNLE

AcHsTRPA --------------------------------------MYENDRNS--LGE-SIKVKNFE

. :

NvHsTRPA LNRAPRSPRSECRKPRHYSLGCIYERQRRTSRDLLPGRLASLYKS-RQGMRPSICPDKVV

MrHsTRPA TQTLRLP-VAHEGRTKHC---SMSDLNDR-SNDLLSTRLASTFRSVR--YRQSSNSEKIV

AmHsTRPA TQTLRVP-LSREGRTIHC---SLSNLNDP-SNDLLSTRLTSTFRSVR--YRQSSNSEKIA

BtHsTRPA TQTLRLP-LPGEGKTIHC---SLSNLNDP-SNDLLSTRLAS-FRSVR--NRQSSSSEKIV

HsHsTRPA LDLLKVPIVSERDQHTYC--SGSLN-SRL-KDEPLAVRLASTYRSLRYIYDRGDNPRRVA

CfHsTRPA TNTLRLPIILDEGRQRLY---SSFN-NHR-KDDSLSVRLASTYRSLRCAYSPEDCSQKIT

LhHsTRPA TGILKVPVILDESRHHIY--TRSFINSRH-KDDSLTARLASTYRSLRCTYATEDYPQKIT

PbHsTRPA MTTLRIPPVLND-EQHFCSTSRSFN-SRH-KNDPLSVRLASTYRSLRSAYAAGDYPEKSA

SiHsTRPA INSLKIPTILNDGEQHFY--SRSFN-SRH-KRDPLSIRLASTYRSLRCTYAAKDYPQKIM

AeHsTRPA TNNLETPTILND-EEHFY--SKSFNKSCH-KSDPLSVRLTPTYRSLRYAY-AEDYPQKIV

AcHsTRPA TNSLETLTILND-EEHFY--SKSFN-SHH-KSDPLSVRLAPTYRSLRCAY-AEDYPQKIV

. . : *. **:. :.* * . .

NvHsTRPA V----TEPQDSAEHVEIDAGAPPPVDDSLFFDRE--FEALKHVKIDGTSLKTAMWAAVNV

MrHsTRPA TNDA-PLITTTNDHVEIDAGTPPPVDESLFYDN---LDVLRQAQINSNDLRSIMWSIVTT

AmHsTRPA IDDI-PPADSSNDHVEIDAGTPPPVDESLFYDN---LDVFRQAQINSNDLRSIMWSIVTT

BtHsTRPA IDDI-PLIETTNDHVEIDAGTPPPVDESLFYDN---LDVFKQAQINSNDLRSIMWSIFTT

HsHsTRPA SGDG-AAAEANAGQLEIDAGEPPPADESLFFDN----GECRHPYVNGARLRSAIWSIVSP

CfHsTRPA IDGVRSASESDSKQIEIDAGVPPPADESLFFDN---LECCKNHYVNGTKLRSAIWSIVDP

LhHsTRPA IGDA-TMLESNNDQIEIDAGEPPPADESFFFDN---LKYCKNHHMNGAKLRYAIWSIVDP

PbHsTRPA INDI-TVPKFDNDGIEIDAGEPPPVDDSLFFDN---LECCKNHYVNGARLRSAIWSIVDS

SiHsTRPA IDDV-TVSKFDNDQIEIDAGEPPPIDDSLFFDN---LECCKNHYVNGAKLRSVLWSIVDS

AeHsTRPA IDDA-TI--FDNDQIEIDAGVPPPVDDSLFFDNLEILECHKNHYVNGAKLRSAIWSIVDT

AcHsTRPA IDDA-TI--FDNDQIEIDAGVPPPADDSLFFDNLEILECYKNHYVNGAKLRSAIWSIVDM

. :***** *** *:*:*:*. .: ::. *. :*: .

**AR1**

NvHsTRPA VEMKDLLEMEKGNIEKGPDGKLLPNA--DQRFKNVMFLWACFRGLAHLLPKLEECGADRD

MrHsTRPA AEMKLLLELEKCNA--------LPEMPPGERIRNIAYLWSCYRGLVHLLPKLEQRGVKCE

AmHsTRPA AEMKILLQLEKCNA--------LPEIPAGERIRNIAYMWCCYRNLAHLLPKLEQSGVKLD

BtHsTRPA AEMKILLELEKCNT--------LPAMPPGERMRNIAYMWSCYRGLVHLLPKLEQSGARHD

HsHsTRPA AETRLLLHMEKCDV--------LPSACKSERLRNVAYIWAAYRGLLQLLPQLEQAGARHD

CfHsTRPA TETRLLFDMEKNRA--------LPDACKSERLRNVAYVWASYRGLLHLLPELENAGAHYD

LhHsTRPA AETKLLLQMEKSSV--------LPNACKDDRLRNVAYIWASYRGLLHLLPELENAGARYD

PbHsTRPA AETRLLLQMEKNRA--------LPRACKSEKLRSVAYIWASYRGLLHLLPELENAGARCD

SiHsTRPA TETRLLLHMEKSRM--------LPNACKSERLRNVAYIWASYRGLLHHLPELENAGAHYN

AeHsTRPA METRLLQYMEKSRT--------LPNACKSENLRNVAYIWASYRGLLHLLPELENAGARYD

AcHsTRPA METRLLQYMEKSRT--------LPNACKSENLRNVAYIWASYRGLLHLLPELEDAGAHYD

* . * :** ** .:.:..: ::*..:*.* : **:**: *. :

**AR2 AR3**

NvHsTRPA YVEPCTGMNPILVASLTSSIACLEYLIEKGADVNYANTALFYTPLHFAAFGNSSEAAELL

MrHsTRPA YVEPCTGMNAILAASLSGNVSCIKHLIKKGVDINCRNPINYYTPLHFAVLGNSTDAARVL

AmHsTRPA YVEPSTGMNAILAASLSGSVACIEHLIKRGVDINYRNPINHYTPLHFAILGNSPDTARIL

BtHsTRPA YVEPCTGMNAILAASLSGNVACIEHLIKRGVDINYRNPINHYTPLHFSVLGNSPDVARIL

HsHsTRPA YVESRSGVNALLAASLGDKVACVEYLIKKGADVNYENLINHYTILHVVALGNSWHAAATL

CfHsTRPA YVESRTGMNAILAASLGNRVDCVEYLIKRGADVNYESFITHFTPLHFAALGNNWQVATTL

LhHsTRPA YVEPRTGVNAILAASLGDKVACVEYLISKGADVNYESSITRYRPLHFAALGNSWHAANTL

PbHsTRPA YVEPRTGVNAILAASLGDKLACVEYLIEKGADVNYESSITRYTPLHFAALGNSWHAAATL

SiHsTRPA YVEPRTGVNAILAASLGDKVACVEYLIAKGADVNYESLITRYTPLHFAALGNSWHTAATL

AeHsTRPA YVEPRTGVNAILAASLGDKVACVEYLIAKGANVNYESPIIHYTPLHFAALGNSWHAAATL

AcHsTRPA YVEPRTGVNAILAASLGDKVTCVEYLIAKGANVNYESPIIHYTPLHFAALGNSWHAAATL

***. :*:*.:*.*** . : *:::** .*.::* . : **. :**. .* *

**AR4**

NvHsTRPA IKHGAKLNSSNC-QD-VEPVLHCAVRARAEKVVKLLLEKAASVAQKNSTGETPLHVACFV

MrHsTRPA LDNGAKPSTYLY-QEVVEPVLHCAIRAGTVEIVKLLLDRGASVVEKNHTGETPLHVACFV

AmHsTRPA LDNGAKPSTYLY-QEVAEPVLHCAIRAGAVEIVKLLLERGASVVEKNHMGETPLHVACFV

BtHsTRPA LDNGAKPSTYLY-QETVEPVLHCAIRAGAVKIVKLLLEQGASVVEKNHMGETPLHVACFI

HsHsTRPA LDYGARLN-YSGQQEMVEPILHSAVRAKAVETVTVLLERGASVVEKNHLGQTPLHVACSV

CfHsTRPA LNNGAKLN-YIC-QEVIEPVLHSAVRAKAVETVRLLLEHGASVVEKNHLGQTPLHVACFV

LhHsTRPA LDNGAKLN-YVC-QEVVEPVLHSAVRAKAVKTVKLILDRGASVVEKNHLGQTPLHVACFV

PbHsTRPA LDHGAKLN-YVC-QGVVEPVLHSAVRAKAVETVRLLLERGASVVEKNHLGQTPLHVACFV

SiHsTRPA LDHGAKLN-YVC-QEVVEPILHSAVRAKAVETVKLLLERGASVVEKNHLGQTPLHVACFV

AeHsTRPA LDHGAKLN-YVC-QEVVEPILHSAIRAKAVETVKLLLERGASVVEKNHLGQTPLHVACFV

AcHsTRPA LDHDAKLN-YVC-QEVVEPILHSAIRAKAVETVKLLLERGASVVEKNHLGQTPLHVACFV

:. .*. . * **:**.*:** : : * ::*:..***.:** *:******* :

**AR5 AR6**

NvHsTRPA QSIGCTELLLCSPGTD--PNAVDQNHRTPLHYAVMNTCSAPELVELLLKHGAAVNVKDKQ

MrHsTRPA QSIECVELLLESPGTN--INAVDRAHRTPLHFAVMTTHSSAKLVELLLKHGALINAADKT

AmHsTRPA QSIKCVELLLDSPGTN--VNAVDRAHRTPLHFAVMTTYSSAKLVELLLKHGALVNAADKT

BtHsTRPA QSIKCVELLLDSPGTN--INAVDRAHRTPLHFAVMTTYSSAKLVELLLKHGALVNAADKT

HsHsTRPA QSMACVELLSAGAAVANIINAVDREYRTPLHFAVMSTDSSVELVQLLLKRGALVNAADKT

CfHsTRPA QSIPCVELLLSSMPTS--VNAVDREHRTPLHFAVMSTDSSIELVQLLLKHGVLVNAADRT

LhHsTRPA QSLPCVELLSAGAS-N--VNDVDRELRTPLHFAVMSTDSSAELVQLLLKRGALVNAADRT

PbHsTRPA QSIPCVELLSASAP-N--VNAVDREHRTPLHFAVMSTASSAELVQLLLKRGALVNAIDRT

SiHsTRPA QSIPCVELLSASAP-N--VNAVDRELRTPLHFAVMSTDSSAELVQLLLKRGALVNAVDRT

AeHsTRPA QSIPCVELLSASAP-N--VNAVDREHRTPLHFAVMSTDSSAELVQLLLKCGALINAADRT

AcHsTRPA QSIPCVELLSASAP-N--VNAVDREHRTPLHFAVMSTDSSVELVQLLLKCSALINAADRT

**: *.*** . * **. *****:***.* *: :**:**** .. :*. *.

**AR7**

NvHsTRPA ELTPLHIASLNEQSQCVDALIWAGADVSATTKTGLTALNIILRKIPESLQVFRQRLDASI

MrHsTRPA GFTPLHVAALNEQSHCVDVLIWAGADVSATTSAGLSALNIILRKIPDSLQVFRQRLDASI

AmHsTRPA GFTPLHVAALNEQSHCVDVLIWAGADVSATTSAGLSALNIILRKIPDSLQVFRQRLDASI

BtHsTRPA GFTPLHVAALNEQSHCVDVLIWAGADVSATTSAGLSALNIILRKIPESLQVFRQRLDASI

HsHsTRPA GFTPLHIAALNEQSRCVDALIWAGADISATTCTGLSALNVILKKIPESLHVFRQRLDASI

CfHsTRPA GFTPLHIAALNEQSRCVDALIWAGADISATTCTGLSALNVVLKKIPESVHVFRQRLDASI

LhHsTRPA GFTPLHIAALNEQSRCVDALIWAGADISATTCTGLSALNIILKKIPESLHIFRQRLDASI

PbHsTRPA GFTPLHIAALNEQSRCVDALIWAGADISATTCTGLSALNIILKKIPESLHVFRQRLDASI

SiHsTRPA GFTPLHIAALNEQSRCVDALIWAGADISATTRTGLSALNIILKKIPESLHVFRQRLDASI

AeHsTRPA GFTPLHIAALNEQSQCVDALIWAGADISATTCTGLSALNIILKKIPESLHVFRQRLDASI

AcHsTRPA GFTPLHIAALNEQSQCVDVLIWAGADISATTCTGLSALNIILKKIPESLHVFRQRLDASI

:****:*:*****.***.*******:**** :**:***::*.***:*:::*********

NvHsTRPA RLKRPVPHNREFEMRLHFDLLFPSNNQCETSFINTFVQEHQKDLLSHPLVMAFLHLKWEK

MrHsTRPA TLRRPVPHNREFEMRLHFDLLFPSGNQCETSFINTFVQERCKDLLSHPLVMAFLHLKWEK

AmHsTRPA TLRRPVPHNREFEMRLHFDLLFPSGNQCETSFINTFVQERRKDLLSHPLVMAFLHLKWEK

BtHsTRPA TLRRPVPHNREFEMRLHFDLLFPSGNQCETSFINTFVQERRKDLLSHPLVMAFLHLKWEK

HsHsTRPA RLTRPVSHNREFEMKLHFDILFPSNNQCETSFINTFVQEHRKDLLSHPLVMAFLHLKWEK

CfHsTRPA RLTRPVSHNREFEMRLHFDILFPSNSKCETSFINTFVQEHRKDLLSHPLVMAFLHLKWEK

LhHsTRPA RLTRPVSHNREFEMRLHFDILFPSNNKCETSFINTFVQEHRKDLLSHPLVMAFLHLKWEK

PbHsTRPA GLTRPVPHNREFEMRLHFDILFPSGNRCETSFINCFVREHRKDLLSHPLVMAFLHLKWEK

SiHsTRPA RLTRPVSHNREFEMRLHFDILFPSGNQCETSFINTFVQEHRKDLLSHPLVMAFLHLKWEK

AeHsTRPA RLTRPVSHNREFEMRLHFDILFPSGNQCETSFINTFVQERRKDLLSHPLVMAFLHLKWEK

AcHsTRPA RLTRPVSHNREFEMRLHFDILFPSGNQCETSFINTFVQEHRKDLLSHPLVMAFLHLKWEK

* ***.*******.****:****...******* **.*. *******************

**S1**

NvHsTRPA IRKFYLMRIFLYALTVIVMTTYVLTALAYRCYNLS-ESKSSKICNNNRASGFLFRRHVIE

MrHsTRPA IRKFYLLRIMLYAMTVICMTTYVLTALAYKCYNYD-DASSSKICSSKRISGFLFRRPVIE

AmHsTRPA IRKFYLLRILVYAMTVICMTTYVLTALAYKCYNHD-EANSSKICSSKRISGFLFRRPVIE

BtHsTRPA IRKFYLLRILVYAMTVICMTTYVLTALAYKCYNYD-EANSSKICSSKRISGFLFRRPVIE

HsHsTRPA IRKVYLLRIFLYAMTVICMTTYVLTALAYKCYNH--ETNNSKICSGKKIS-FLFRRPVIE

CfHsTRPA IRKLYLLRIFLYAMTVICMTTYVLTALAYKCYNHEVTTDSPKICDSKRAVDFLFRKPFIE

LhHsTRPA IRKLYLLRIFLYALTVICMTTYVLTALAYKCYNNN-ETNSSKICSSKRAADFLFRKPVIE

PbHsTRPA IRKLYLLRILLYTMTVICMTTYVLTALAYKCYNYN-DANSSKICGSKRASAFLFRRPIIE

SiHsTRPA IRKLYLLRILLYAMTVICMTTYVLTALAYKCYNQN-DTNSTRICGSRRVSGFLFRRPIIE

AeHsTRPA IRKLYLLRIFLYSMTVICLTTYVLTALAYKCYNQN-ETNSSKICGSRRPSDFFFRKSIIE

AcHsTRPA IRKLYLLRILLYSMTVICLTTYVLTALAYKCYNQN-EANSSKICGSRRPSDFFFRKSIIE

***.**:**::*::*** :**********.*** :....**.... *:**. .**

**S2 S3**

NvHsTRPA IEWYVSLILTCVTIPRKIFGFMVYKSARQYFMNIDNVLDAIVIVSVFVTSFIYTGRTYDW

MrHsTRPA IQWYLLFIFTCISIPRKIFGFMVYTSAKQYFSNIDNVLDGVVIISVFVTSFVYTGRTYDW

AmHsTRPA IQWYLLLIFTCISIPRKIFGFMVYTSAKQYFSNIDNVLDGVVIISVFVTSFVYTGRTYDW

BtHsTRPA IQWYLLLIFTCISIPRKIFGFMVYTSAKQYFSNIDNVLDGIVIISVFVTSFVYTGRTYDW

HsHsTRPA IEWYLALALTCITIPRKVIGFMVHKSAIQYFSNIDNVLDGVVIVSIFVTSFIYTGRTYDW

CfHsTRPA IQWYLALVLTCITIPRKIFGFMVYKSALQYFINIDNILDGVVIISVFVTSFIYTGRTYDW

LhHsTRPA IEWYLSLALTCITIPRKIFGFMVYRSMIQYFSNIDNVLDAVMIMSVFVTSFIYTGRTYDW

PbHsTRPA VEWYLALVLTCITIPRKIFGFMVYKSAIQYFSSIDNVLDGVVIVSVFVTSFVYTGRTYDW

SiHsTRPA IEWYLALVLTCITIPRKIFGFMVYKSAIQYFSNIDNVLDGVVIVSVFVTSFVYSGRTYDW

AeHsTRPA IEWYLALVLTCITIPRKIYGFMVYKSALQYFSSIDNVLDGVVIVSVFVTSFVYTGRTYDW

AcHsTRPA IEWYLALVLTCITIPRKIYGFMVYKSALQYFSSIDNVLDGVVIVSVFVTSFVYTGRTYDW

::**: : :**::****: ****: * *** .***:**.::*:*:*****:*:******

**S4 S5**

NvHsTRPA QNYVGAFAILCAWTNLMLMVGQLPAFGTYVAMFTHIQFEFAKLLLAYSGLLIGFTISFCV

MrHsTRPA QNYVGAFAILCAWTNLMLMVGQLPAFGTYVAMFTHIQFEFAKLLLAYSGLLIGFTVSFCV

AmHsTRPA QNYVGAFAILCAWTNLMLMVGQLPAFGTYVAMFTHIQFEFAKLLLAYSGLLIGFTISFCV

BtHsTRPA QNYVGAFAILCAWTNLMLMVGQLPAFGTYVAMFTHIQFEFAKLLLAYSGLLIGFTISFCV

HsHsTRPA QNYVGAFAILCAWTNLMLMVGQLPAFGTYVAMFTHIQFEFAKLLLAYSGLLIGFTISFCV

CfHsTRPA QNYVGAFAILCAWTNLMLMVGQLPAFGTYVAMFTHIQFEFAKLLLAYSGLLIGFTISFCV

LhHsTRPA QNYIGAFAILCAWTNLMLMVGQLPAFGTYVAMFTQIQFEFAKLLLAYSGLLIGFTISFCV

PbHsTRPA QNYVGAFAVLCAWTNLMLMVGQLPAFGTYVAMFTHIQFEFAKLLLAYSGLLIGFTVSFCV

SiHsTRPA QNYVGAFAILCAWTNLMLMVGQLPAFGTYVAMFTHIQFEFAKLLLAYSGLLIGFTISFCV

AeHsTRPA QNYVGAFAVLCAWTNLMLMVGQLPAFGTYVAMFTHIQFEFAKLLLAYSGLLIGFTISFCV

AcHsTRPA QNYVGAFAVLCAWTNLMLMVGQLPAFGTYVAMFTHIQFEFAKLLLAYSGLLIGFTISFCV

***:****:*************************:********************:****

**SF**

NvHsTRPA IFVGEPSFGNPFTGLIKVLAMMAGELDFEGLINQ-DDIL---HDGSFVLYHP-LSVCSQI

MrHsTRPA IFVGEPSFGNPFTGLIKVLAMMAGELDFDSLITQIDQGLE--SEGPFVIYHP-LSVCSQI

AmHsTRPA IFVGEPSFGNPFTGLIKVLAMMAGELDFEGLITQIDQGLE--SDGPFVIYHP-LSVCSQI

BtHsTRPA IFVGEPSFGNPFTGLIKVLAMMAGELDFEGLITQIDEGLEGNSEGPFVIYHP-LSVCSQI

HsHsTRPA IFSGEPAFGNPFTGLIKVLAMMAGELDFEGLINQSDEPA----TGSFVIYHP-LSVCSQI

CfHsTRPA IFAGDPAFGNPFTGLIKVLAMMAGELDFEGLINQADSSST--TGGSFIIYHP-LSVCSQI

LhHsTRPA IFAGEPAFGNPFTGLIKVLAMMAGELDFEGLINQTDDGS---TDKSFVIYHP-LSVCSQI

PbHsTRPA IFAGEPAFDNPFTGLIKVLAMMAGELDFEGLINQVDDEP---TGGPFVIYHP-LSVCSQI

SiHsTRPA IFAGEPAFGNPFTGLIKVLAMMAGELDFEGLLNQMDDEP----TGSFVFYHP-L---SQI

AeHsTRPA IFASEPAFGNPFTGLIKVLAMMVGELDFEGFINQMDDKP---MGGSFVIYHPILSVCSQI

AcHsTRPA IFASEPAFGNPFTGLIKVLAMMVGELDFEGFINQMDDKP----GGSFVIYHPILSVCSQI

** .:*:*.*************.*****:.::.* *. .*::*** * ***

**S6**

NvHsTRPA LFTLFIVFVTVILMNLLVGIAVHDIQGLRNHAGLTKLVRQTKLILFTEMVLYNGRIPYTF

MrHsTRPA LFTLFIVFVTVILMNLLVGIAVHDIQGLRNHAGLTKLVRQTKLILFTEMVLHNSSIPYAF

AmHsTRPA LFTLFIVFVTVILMNLLVGIAVHDIQGLRNHAGLTKLVRQTKLILFTEMVLHNSSIPYAF

BtHsTRPA LFTLFIVFVTVILMNLLVGIAVHDIQGLRNHAGLTKLVRQTKLILFTEMVLHNSSIPYAF

HsHsTRPA LFTLFIVFVTVILMNLLVGIAVHDIQGLRNHAGLTKLVRQTKLILFTEMILHNAKIPYAF

CfHsTRPA LFTLFIVFVTVILMNLLVGIAVHDIQGLRNHAGLTKLVRQTKMILFTEMVLHNTTIPYAF

LhHsTRPA LFTLFIVFVTVILMNLLVGIAVHDIQGLRKHAGLTKLVRQTKMILFTEMVLHNTSIPYAF

PbHsTRPA LFTLFIVFVTVILMNLLVGIAVHDIQGLRNHAGLTKLVRQTKMILFTEMVLHNTTIPYAF

SiHsTRPA LFTLFIVFVTVILMNLLVGIAVHDIQGLRKHAGLTKLVRQTKMILFTEMVLHNTTIPYAF

AeHsTRPA LFTLFIVFVTVILMNLLVGIAVHDIQGLRNHAGLTKLVRQTKMILFTEMVLHNTAIPYAF

AcHsTRPA LFTLFIVFVTVILMNLLVGIAVHDIQGLRNHAGLTKLVRQTKMILFTEMVLHNTAIPYAF

*****************************:************:******:*:* ***:*

NvHsTRPA KKWMSDHKIDVDNRKRVLVVKPLNPLEKRLPKDIMKAAYEIAQRNAPLVDEDDVSLDEHV

MrHsTRPA RKWMSDHKINVENRRRVLVVKPLNPLEKRLPKDILKAAYEIAQKNIPFMNDDNISLSDHV

AmHsTRPA RKWMSDHKINVENRRRVLVVKPLNPLEKRLPKDILKAAYEIAQKNIPFMNDENINLSDHV

BtHsTRPA RKWMSDHKINVENRKRVLVVKPLNPLEKRLPKDILKAAYEIAQKNIPFMNDDNINLSDHV

HsHsTRPA RKWMSDHKIDVENRKRVLVVKPLNPLEKRLPKDILKAAYEIAQKNIP-FMNDDVSLSDHV

CfHsTRPA RKWLSDHKIDVENRKHVLVVKPLNPLEKRLPKDIMKAAYEIAQKNIP-LANDDVNLNDHA

LhHsTRPA RKWLSDHKIDVENRKRVLVVKPLNPLEKRLPKDIMKAAYEIAQKNVP-YANDDVHLSDYA

PbHsTRPA RKWMSDHKIDVENRKRVLVVKPLNPLEKRLPKDIMKAAYEIAQKNIP-FVNDDINLNDHA

SiHsTRPA RKWMSDHKINVENRKRVLVVKPLNPLEKRLPKDIMKAAYEIAQKNIPSSANDDINLSDHA

AeHsTRPA RKWMSDHKIDVENRKRVLVVKPLNPLEKRLPKDIMKAAYEIAQKNIPSFMNDDINLSDRA

AcHsTRPA RKWMSDHNIDVENRKQVLVVKPLNPLEKRLPKDIMKAAYEIAQKNIPSFIND-INLNDRA

.**:***:*:*:**..******************:********.* * :: : *.: .

NvHsTRPA TWMK-QQSEEGSDYLLHSTIDQLSTQMKSAEDDIKVIKEQLLDTNKMLKNLVARRVQPR-

MrHsTRPA MWMR-QQSEEYSDSSLQLVIENLANKLQSYEDTIKSLKEQLLDTNKTLEGIVKSLTKEKS

AmHsTRPA IKMR-QHSEEYFDSNLQLVIENLANKLQSYEDMIKSLKEQLLDTNKMLESVVKNLAKEKN

BtHsTRPA MRMR-QQSEDCFDSNLQLVIENMANKLQSYEDTIMLLKEQLLDTNKMLESVVKNLTKEKN

HsHsTRPA AWVK-RQNEELSDAAVHMAIERLVTTVKMNEDAVKLLKVELMEMNKMLKTLITTLARGRT

CfHsTRPA AWLKQRQNNEVSDAVLQTTIEKLITAMKVNEDAVKLLRVQLLEMNKMLETIVATLAKEQY

LhHsTRPA TWLKQRRSEEYSDAA-QIAIEKLIAMMKVNEDAIKLLRMQMLEMNKMLENVVTTLEKEEH

PbHsTRPA IWLKQRQNEEFSDIALQMTFEKLIAMMKVNEDAIKLLKIQLLEMNKMLETITITLTKEER

SiHsTRPA VWLK-HRQAEFSDVALQMTFEKLISMMKVNEDAIKLLRVQLLEMNKMLENVVITLAKEEN

AeHsTRPA VWLK-QRHNGFSDGALEMTFEKLIAMMKVNEDAIKLLRIQLLEMNKMLENVATKLTKEEH

AcHsTRPA VWLK-QRHNEFSDVAPEMTFEKLIAMMKVNEDAIKLLRVQLLEMNKMLENIATKLTKEEH

.:. .. * .::.: :: ** : :. :::: ** *: : .

NvHsTRPA ----

MrHsTRPA N---

AmHsTRPA N---

BtHsTRPA N---

HsHsTRPA SS--

CfHsTRPA PSL-

LhHsTRPA NSL-

PbHsTRPA TSL-

SiHsTRPA TSL-

AeHsTRPA TTSL

AcHsTRPA TASL
