# Supplementary figures and images for: HsTRPA of the Red Imported Fire Ant, Solenopsis invicta, Functions as a Nocisensor and Uncovers the Evolutionary Plasticity of HsTRPA Channels
Source: eNeuro. 2018 Feb 6;5(1):ENEURO.0327-17.2018. doi: 10.1523/ENEURO.0327-17.2018 (PMC5810042; doi:10.1523/ENEURO.0327-17.2018)

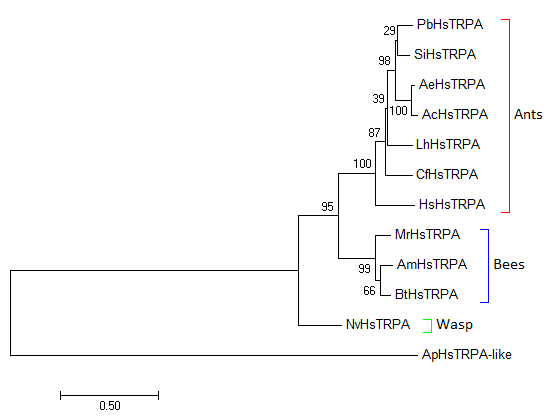

Supplement: Figure 6-2 — Molecular phylogenetic tree of wasp, bee, and ant HsTRPA channels. The evolutionary history of wasp, bee, and ant HsTRPA channels was inferred by using the maximum likelihood method based on the JTT matrix-based model. A discrete gamma distribution was used to model evolutionary rate differences among sites. The tree is drawn to scale, with branch lengths measured in the number of substitutions per site. Acyrthosiphon pisum (pea aphid) HsTRPA-like channel (ApHsTRPA-like) was used as the outgroup. Bootstrap values with 500 replications are also shown at each node of the tree. Ant, bee, and wasp HsTRPA channels are indicated by red, blue, and green brackets, respectively. Download Figure 6-2, TIF file. [file sup_enu-eN-NWR-0327-17-s09.tif]

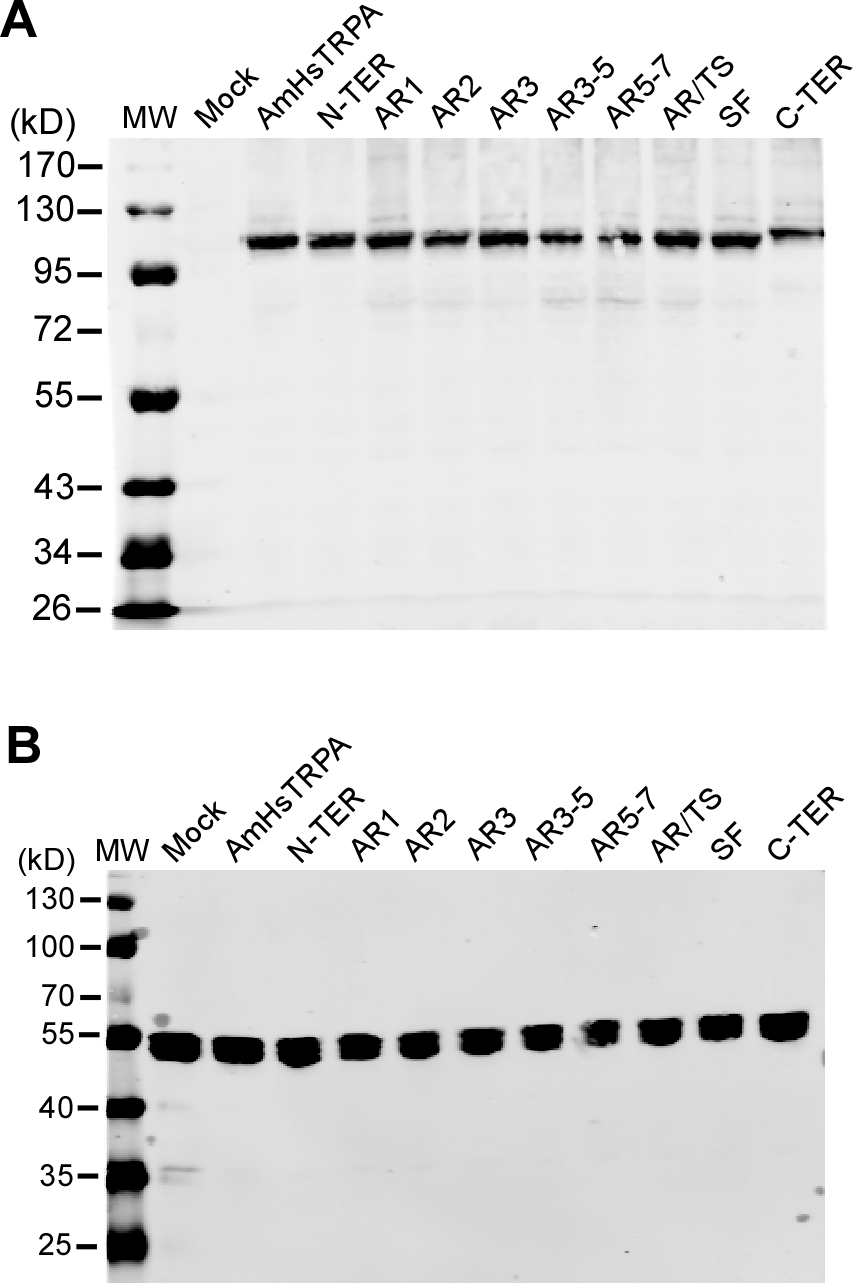

Supplement: Figure 7-3 — Expression of nine AmHsTRPA/SiHsTRPA chimeric channel proteins. The channel proteins (A) and β-tubulin (B) expressed in HEK293 cells transfected with empty vector (Mock), AmHsTRPA- and AmHsTRPA/SiHsTRPA chimeric channel (N-TER, AR1, AR2, AR3, AR3-5, AR5-7, AR/TS, SF, and C-TER)-expressing constructs were analyzed by Western blot. The size (in kilodaltons) of protein molecular weight marker (MW) is at the left. Download Figure 7-3, TIF file. [file sup_enu-eN-NWR-0327-17-s10.tif]
